# A one-step volumetric biofabrication platform for complex hydrogel-based hollow biomaterials

**DOI:** 10.1016/j.mtbio.2026.103594

**Published:** 2026-08-28

**Authors:** Luís P.G. Monteiro, João M.M. Rodrigues, João F. Mano

**Affiliations:** CICECO – Aveiro Institute of Materials, Department of Chemistry, University of Aveiro, Aveiro, 3810-193, Portugal

**Keywords:** Biofabrication, Rotational molding, Hydrogels, Tissue engineering, Proteins, Polysaccharides

## Abstract

Rotational molding (RM) is a widely used industrial process for producing hollow polymeric structures, yet it remains largely unexplored for biomaterials fabrication. Here, a low-cost 3D-printed biofabrication platform is introduced that adapts the general concept of RM to generate tunable hollow constructs with high structural fidelity. Using uniaxial RM, tubular-like hydrogels with precisely controlled diameter and wall thickness are generated by crosslinking light-responsive natural-based polymers, such as modified proteins and polysaccharides, under mild, cell-compatible conditions. The process yields reproductible geometries and well-defined walls while maintaining long-term cell viability. Furthermore, extending the system to biaxial RM allows the rapid, one-step fabrication of more complex hollow 3D architectures. By integrating established RM principles with biocompatible photochemistry, this accessible platform provides a scalable and versatile route to engineer hollow hydrogel architectures using virtually any kind of hydrogel forming material, independent of their rheological properties or crosslinking conditions. These capabilities expand opportunities in tissue modeling, biohybrid living actuators, and regenerative medicine, positioning RM as a powerful strategy for the controlled design of functional hollow hydrogels.

## Graphical abstract

**Figure:**
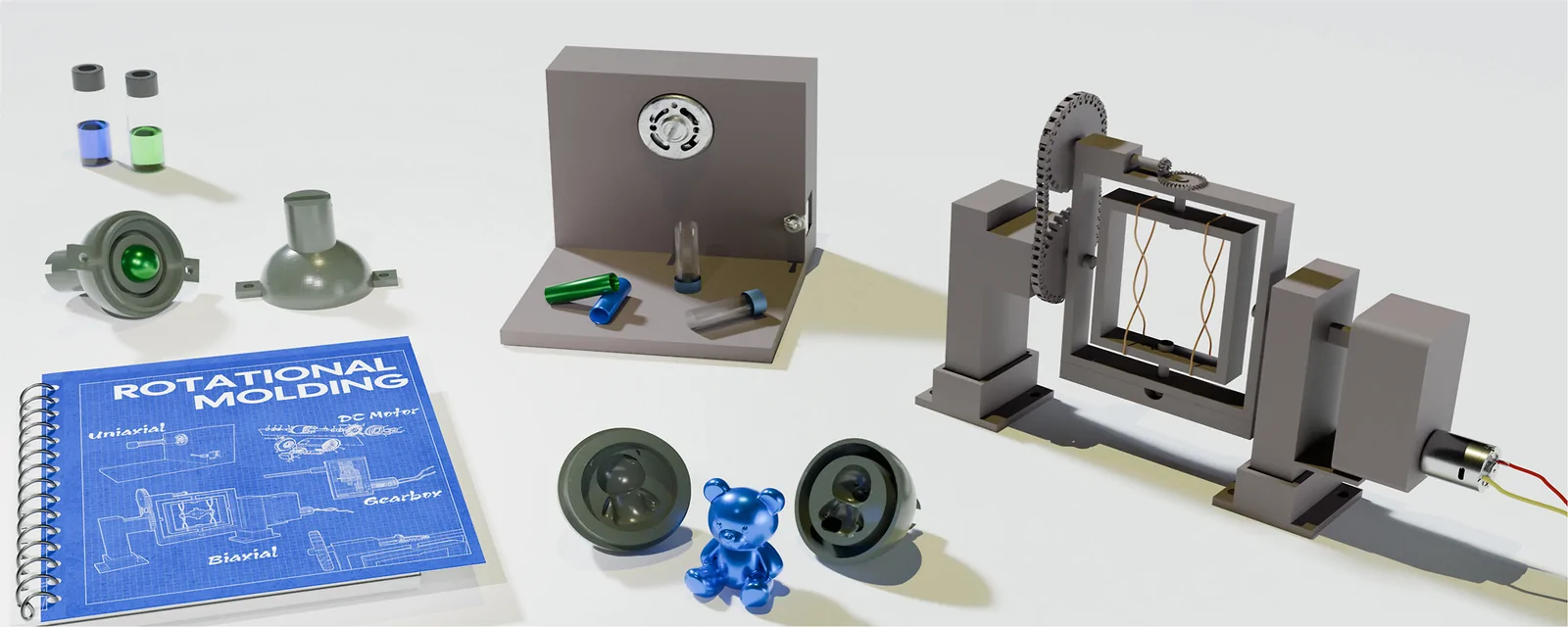

## 1 Introduction

Additive manufacturing has advanced rapidly over the past two decades, from early demonstrations of cell and organ printing in 2003 [1] to recent developments such as two-photon 3D printing within living cells [2] and adaptive context-aware volumetric printing [3]. Despite the broad range of available biofabrication techniques [4,5], including laser-assisted bioprinting and drop-on-demand methods, the field remains largely dominated by extrusion-based bioprinting [6,7]. In this technique, bioinks are dispensed through fine nozzles using pneumatic or mechanically driven forces and deposited as continuous filaments in a computer-guided, layer-by-layer process [6,8]. In 3D bioprinting, a “one-size-fits-all” strategy is rarely feasible, as the process is inherently multifactorial [9] and requires careful balancing of printability with high cell viability. While some materials exhibit favorable rheological properties but provide suboptimal biological environments, others, such as gelatin, collagen, and decellularized extracellular matrix, offer excellent biological performance but poor printability or structural stability [[9], [10], [11]]. Achieving high shape fidelity typically demands optimization of multiple rheological parameters, including ideal viscosity drop, appropriate viscosity recovery after applied shear forces (extrusion), yield stress, self-healing behavior, shear-thinning profile, ideal gelation time and crosslinking kinetics, among others [[10], [11], [12], [13], [14]]. In parallel printing conditions such as pressure and deposition speed, temperature, and nozzle geometry must be finely tuned and have a major impact on the fidelity of the 3D-printed construct [15,16]. Beyond limitations in resolution, extrusion-based 3D bioprinting also struggles with the fabrication of multimaterial constructs and hollow and/or gravity-defying architectures [17], largely because thin walls are mechanically fragile and prone to deformation [18,19]. Finally, the high cost of commercial bioprinting platforms represents a significant bottleneck, motivating the development of open-sources approaches that convert conventional low-cost 3D printers into functional 3D bioprinters [[20], [21], [22], [23]].

Rotational molding (RM) has historically shaped thermoplastics manufacturing by enabling stress-free fabrication of hollow, layerless parts with homogeneous and tunable wall thickness, while also supporting highly complex geometries [24,25]. RM typically involves four steps: (i) charging the mold with polymer, (ii) heating under biaxial rotation, (iii) cooling, and (iv) demolding [26]. Compared with extrusion-based 3D (bio)printing, RM provides a more straightforward approach for producing hollow, multimaterial, and multilayered structures, as controlled stratification is often challenging to achieve by nozzle-based deposition [27]. However, translating RM to biomaterials is nontrivial. Most natural and many synthetic polymers used in biofabrication are thermally unstable, and thermal processing is incompatible with encapsulated living cells. In essence, the general concept of RM can be extended to liquid hydrogel precursors, which solidify via chemical crosslinking within the rotating mold, enabling the fabrication of complex hollow structures without thermal annealing. Diverse chemical strategies can be employed to crosslink polymer precursors into hydrogels [28], and these have been used to generate hollow, vasculature-like architectures via casting into tubular molds [29], sheet-rolling [30,31], layer-by-layer coating on cylindrical molds [32], and multiple 3D-printing methodologies [31,33,34]. While these methods have enabled substantial progress, each presents specific limitations related to scalability, multi-step fabrication process or reliance on layer-by-layer deposition. In contrast, RM provides a layerless fabrication strategy that combines simplicity and cost-effectiveness with the one-step production of hollow constructs. This concept was briefly explored in early studies to construct hollow tubular structures [35]. The concept of “rotational internal flow layer engineering” was later introduced [36] to fabricate hollow tubes by sequential deposition of alginate and Ca^2+^ in uniaxially rotating tubular molds, enabling the fabrication of stratified architectures. However, this approach primarily relies on ionic crosslinking and thermoresponsive systems [37] and does not explore the full potential of RM to engineer more complex three-dimensional structures, thereby limiting its broader applicability.

Here, we present a low-cost RM platform and demonstrate that uniaxial RM can produce stratified hollow tubes with tunable diameter and precise wall thickness. We validate the concept by crosslinking light-responsive biopolymers, under mild conditions compatible with long-term cell viability. We further extend the approach to biaxial RM, where redox-initiated radical polymerization supports the fabrication of more complex hollow 3D architectures. Notably, each setup can be assembled for less than 15 €, with all custom modules fabricated using a conventional 3D printer, highlighting the accessibility of the platforms. By integrating RM principles with biocompatible chemistries, this strategy expands the toolbox for one-step volumetric biofabrication, with potential applications in tissue models, biohybrid living actuators, and regenerative medicine.

## 2 Results and discussion

### 2.1 Single material uniaxial RM: design and construction

**Fig. 1:**
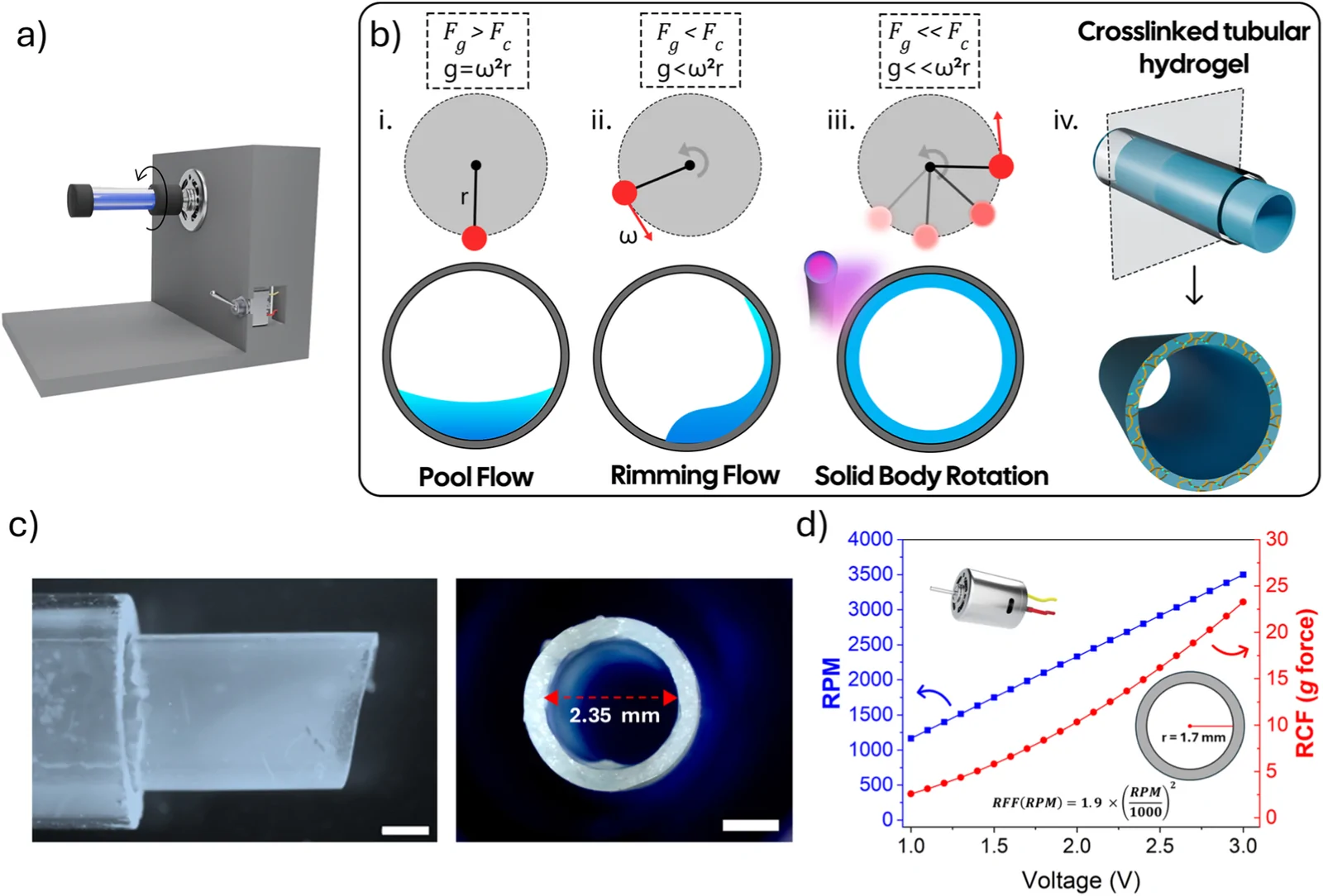
Schematic illustration of the uniaxial RM apparatus (a), comprising a DC motor mounted on a 3D-printed frame and a 5 V electrical circuit connected to a DC–DC converter board with a parallel ON/OFF switch. (b) Main stages of RM: (i) at low rotational speeds, the precursor solution remains pooled at the bottom of the mold; (ii) as rotation increases, centrifugal forces (*Fc*) begin to overcome gravitational forces (*Fg*), resulting in partial coverage of the inner mold surface; (iii) once *Fc* fully exceeds *Fg*, the precursor solution forms a uniform internal coating, after which photocrosslinking (or other crosslinking process) is initiated to yield a (iv) crosslinked tubular construct. c) Digital images of the LamMA-based tubular template with an internal diameter of 2.35 ± 0.012 mm. Scale bar = 1 mm. (d) Predicted motor rotation per minute (RPM) as a function of input voltage (this estimation does not account for power losses), with the corresponding *g*-force calculated based on the internal radius of the glass mold.

The uniaxial RM apparatus is illustrated in Fig. 1a, and its schematic assembly and dimensions are provided in Fig. S1. A 3.5 V DC motor was wired to a DC-DC converter board, enabling precise control of the output voltage and motor rotation speed, and mounted on a 3D-printed frame. Different chemical modification routes can be used to enable polymer crosslinking [28]. Here, laminarin (Lam), a low-molecular-weight natural polysaccharide, was functionalized with photoresponsive groups via methacrylation (LamMA), a widely used approach in biomaterials [[38], [39], [40], [41], [42]]. In the presence of a photoinitiator and light exposure, at an appropriate wavelength, a sol–gel transition is induced within a relatively short time span [38].

**Fig. 2:**
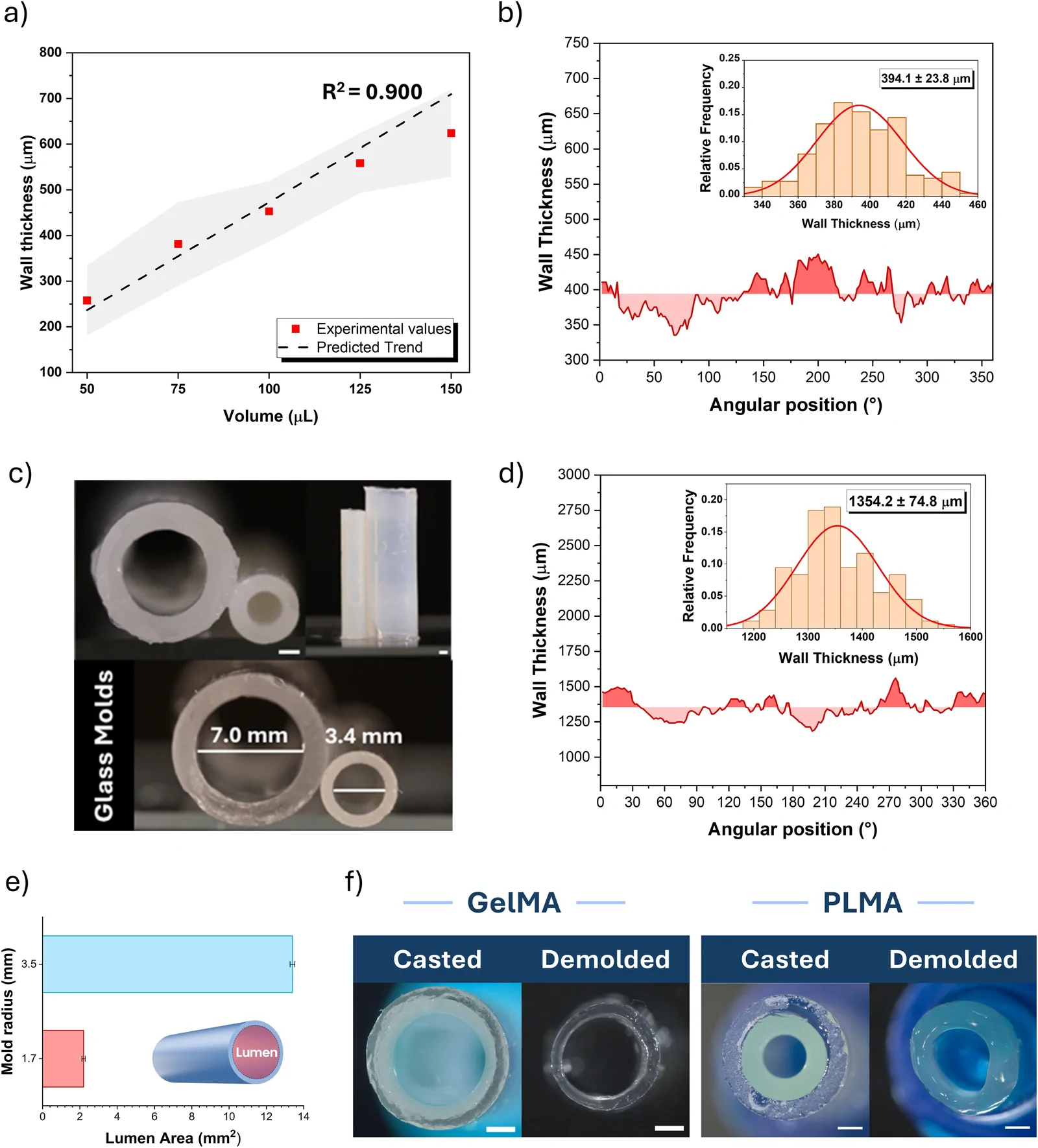
(a) Predicted versus experimental wall thickness of LamMA-based tubular constructs prepared with volumes of LamMA 20% (w/v) ranging from 50 to 150 μL, with the corresponding standard deviation shown in gray (*n* = 3). (b) Wall thickness variation of a tubular template fabricated in a 1.7 mm radius glass mold using 100 μL of 20% (w/v) LamMA. Measurements were acquired at 2° angular increments. Values above and below the calculated average are highlighted in dark and light colors, respectively. The inset shows the corresponding histogram and fitted normal distribution curve (red line). (c) Digital images (top and side view) comparing tubular templates assembled using two different glass molds (3.4 and 7.0 mm inner diameter). Scale bar = 1 mm. (d) Wall thickness variation of a tubular template fabricated in a 3.5 mm radius glass mold using 500 μL of 20% (w/v) LamMA. Measurements were acquired at 2° angular increments. Values above and below the calculated average are highlighted in dark and light colors, respectively. The inset shows the corresponding histogram and fitted normal distribution curve (red line). (e) Cross-sectional area (lumen area) of the tubular constructs corresponding to the two molds (*n* = 3). (f) Stereomicroscope images of tubular templates obtained with methacrylated gelatin – GelMA (100 μL) and human methacrylated platelet lysates – PLMA (150 μL), showing the constructs inside the glass molds and after retrieval. Scale bar = 1 mm. All measurements were performed using ImageJ software.

An aqueous precursor solution containing 20% (w/v) LamMA and 0.5% (w/v) lithium phenyl-2,4,6-trimethylbenzoylphosphinate (LAP) was loaded into a glass mold (1.7 mm internal radius), which was then attached to the motor and sealed with a cap. Once under rotation, centrifugal forces overcome gravity and drive the liquid to conform to the mold's inner geometry. Subsequent photopolymerization fixes the shape (Fig. 1b and Movie S1), yielding a well-defined tubular-like scaffold (Fig. 1c). Since sufficient centrifugal force must be generated to ensure a homogeneous distribution of the precursor mixture, it is also important to keep the resulting shear forces low enough to preserve cell viability during encapsulation [43]. Fig. 1d reports the RPM and relative centrifugal force (RCF, expressed in *g*) calculated as function of the applied voltage (V). Considering the inner diameter of the glass mold, the maximum force generated is approximately 25 *g*, which is unlikely to compromise cell viability. Moreover, since the glass mold constitutes a closed system, the exact rotation speed is not critical as long as a homogeneous liquid distribution is achieved, therefore, all experiments were conducted at 1.5 V (corresponding to ∼ 1750 RPM). Given the hollow cylindrical shape of the glass mold, it is possible to predict the thickness of the resulting hollow tube from the volume of precursor solution. The wall thickness of the hollow constructs can be predicted from the volume of precursor (v) loaded into the mold by approximating the cylindrical geometry as a rectangular prism. Under the assumptions of uniform precursor distribution and negligible material loss, the wall thickness (t) is given by: (t)=v(p*z), where p and z represents the cylinder's circumference and height, respectively. Fig. 2a compared the predicted wall thickness (black dash line) with experimental measurements (red squares), while the gray region indicates the standard deviation. The experimental data closely matches the predicted values, confirming the validity of the geometrical estimation. Using this approach, hollow tubes with wall thickness ranging approximately from 250 to 600 μm were successfully obtained. To further evaluate the effect of processing parameters on wall thickness, an additional experiment was conducted by varying the polymer concentration (15 and 20% (w/v)) and rotation speed (∼1750 and ∼3500 RPM), while maintaining a constant precursor volume. As shown in Fig. S2, neither parameter produced significant changes in the wall thickness, demonstrating that, within the investigated processing window, these variables do not affect the final tube geometry.

To assess thickness homogeneity, a hollow tube was casted using 100 μL of LamMA at 20% (w/v) and analyzed throughout the entire circumference of the tubular hydrogel (Fig. 2b). Thickness measurements revealed that the maximum and minimum wall thickness values deviated from the average (394.1 ± 23.8 μm) by only 14.3% and 14.9%, respectively. This limited dispersion indicates that material distribution during uniaxial RM is largely homogeneous, with no pronounced localized thinning or thickening. The respective histogram further supports wall thickness uniformity, revealing a narrow, unimodal distribution centered around 394.1 μm and well described by a normal distribution. While this analysis demonstrates wall thickness homogeneity within a single cross-section, it does not provide information about homogeneity along the entire length of the tube. Wall thickness measurements were repeated at two additional cross-sectional positions along the tube length. For direct comparison, the dataset presented in Fig. 2b was also included in Fig. S3. Comparable wall thickness distributions were obtained at all three cross-sections, confirming that the uniaxial RM process produces tubular constructs with homogeneous wall thickness throughout their entire length.This behavior suggests that the observed thickness variations are limited and are primarily attributable to process-related fluctuations rather than localized structural defects or systematic asymmetries. Deviations from ideal single-axis rotation would result in uneven wall thickness across the hollow tube (Fig. S4a). In practice, such deviations can arise from slight misalignments or imperfections in the 3D-printed components, particularly the connector between the glass mold and the motor or the tube cap, causing off-axis rotation, ultimately leading to heterogeneous coating (Fig. S4b). Importantly, hollow tubular hydrogels were also successfully fabricated using a larger glass mold (internal radius = 3.5 mm). The successful fabrication of larger LamMA templates, as shown in Fig. 2c, confirmed the scalability of the method. Wall thickness variations in these larger constructs remained limited, with maximum and minimum values deviating from the mean by 15.2% and 12.4%, respectively (Fig. 2d). The corresponding histogram shows a normal distribution, further supporting that wall thickness homogeneity is maintained across different mold sizes. As expected, increasing the mold radius from 1.7 to 3.5 mm resulted in a proportional enlargement of the lumen, with cross-sectional area increasing from approximately 2.2 mm^2^ to 13.4 mm^2^ (Fig. 2e). Collectively, these findings confirm that the proposed RM technique can generate hollow, cylindrical hydrogels whose diameter, lumen area, and wall thickness fall within the physiological range reported for small-to-medium human blood vessels. [44]. Moreover, the intrinsic geometrical scalability of the method suggests that, in principle, RM can produce hollow tubular structures capable of mimicking a wide range of physiological tubular tissues including the esophagus, intestines, trachea, urethra, and others [45].

### 2.2 Multimaterial uniaxial-axis rotational molding

**Fig. 3:**
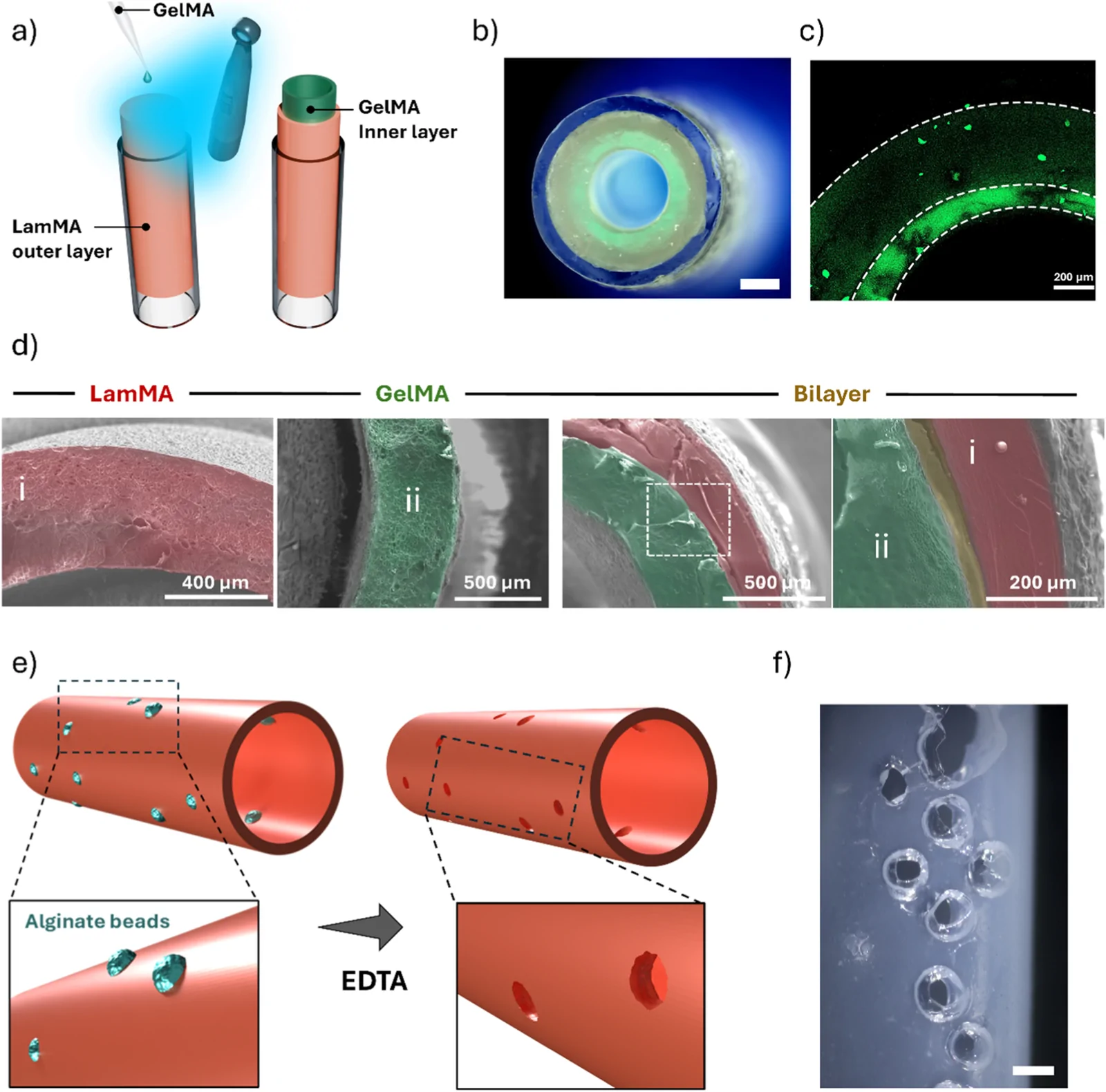
(a) Schematic representation of the procedure employed to design bi-layered tubular templates. (b) Stereomicrograph images of the bi-layered template comprising a GelMA inner layer and a LamMA outer layer. Scale bar = 1 mm. (c) Fluorescence confocal microscopy image of the bi-layered tubular template (top view) with the GelMA layer stained with 6-aminofluorescein. (d) SEM images showing the microstructure of single- and bi-layered constructs. The two layers were labeled with different colors, with i) representing the LamMA layer and ii) representing the GelMA layer. Colors were digitally overlaid. (e) Schematic illustration of the procedure used to customize the wall's topology in a LamMA construct. (f) Stereomicrograph image of a LamMA template after EDTA treatment to liquefy the alginate-Ca^2+^ beads, producing micropores throughout the wall. Scale bar = 500 μm.

The versatility of this fabrication strategy is not limited to polysaccharide-based systems. Owing to its photocrosslinking nature, the method can be readily extended to virtually any methacrylated polymer, including animal- and human-derived methacrylated proteins. Fig. 2f shows stereomicrographs of hollow tubular hydrogels casted from GelMA and PLMA, confirming that the proposed molding technique can be effectively applied to protein-based photocrosslinkable precursors regardless of their viscosity (ranging from 10^−3^ to 10 Pa s – Fig. S5). Beyond demonstrating compositional versatility, uniaxial RM further enables the creation of hollow scaffolds with spatially defined architectures. Taking advantage of this feature, multimaterial, multilayered tubular structures were fabricated by sequentially depositing distinct precursor solutions (Fig. 3a) to better emulate the stratified and hierarchical organization of native hollow tissues. Moreover, this approach enables the fabrication of tubular constructs with layers exhibiting distinct mechanical properties, porosity, and biofunctionality, providing a versatile platform for tuning scaffold features in a spatially controlled manner. A hollow tube comprising an outer layer of LamMA and an inner layer of GelMA was casted to demonstrate the feasibility of multilayered tubular constructs. The outer LamMA layer was fabricated as described above, after which a precursor GelMA solution was poured into the hollow core of the pre-formed LamMA tube. Finally, under centrifugal force, the inner GelMA layer was photocrosslinked. The stereomicrograph images of the bi-layer hollow construct clearly shows the formation of two well-defined layers (Fig. 3b).

To further confirm the presence of the two individual layers, the tubular construct was incubated in a 5 mM solution of 6-aminofluorescein to stain the GelMA layer [46]. Confocal microscopy (Fig. 3c) revealed that the two layers remain largely independent. The fluorescent dye predominantly stained the GelMA layer, however, low levels of fluorescence were also detected within the LamMA layer, likely resulting from partial diffusion of the GelMA precursor solution into the LamMA hydrogel, facilitated by the centrifugal forces applied prior to GelMA photocrosslinking. Complementary SEM analysis (Fig. 3d) corroborated these observations, revealing a distinct interface (highlighted in yellow) between the two polymer-based layers. When compared to their single-layer counterparts, both GelMA and LamMA layers appeared less porous in the bilayer configuration, although this effect was considerably more evident for outer LamMA layer. The two consecutive photocrosslinking cycles experienced by LamMA, together with the additional centrifugal forces acting on this layer during the subsequent deposition of the GelMA layer, may account for the observed morphological differences between the single and bilayer templates.

Uniaxial RM can also be used to tailor wall topology. This can be accomplished by embedding sacrificial materials within the precursor mixture, which, upon removal, create predefined structural features. Following this concept, sacrificial alginate-Ca^2+^ beads (Ø 874 ± 56 μm) were introduced into the LamMA precursor solution and subsequently subjected to RM. The centrifugal forces pushed the beads against the glass mold, incorporating them into the wall of the construct (Fig. S6). Following photocrosslinking and retrieval of the tubular template, the sample was incubated in ethylenediaminetetraacetic acid (EDTA) to liquify the alginate beads, leaving behind their negative imprints, as illustrated in Fig. 3e. As shown in Fig. 3f, EDTA treatment and consequent alginate-Ca^2+^ beads liquification produced microporous throughout the wall of the construct, effectively reproducing the negative imprints of the beads. It is important to note that the successful formation of micropores via sacrificial bead removal relied on the bead diameter being larger than the wall thickness of the tubular constructs. Only beads larger than the wall can span its entire thickness, whereas smaller beads would be fully embedded, and their subsequent dissolution would not produce porosity. This illustrates the capacity of uniaxial RM to produce tubular templates with tailored wall topologies, providing a versatile platform for the design of architecturally sophisticated constructs. Several fabrication strategies have been proposed for the fabrication of hollow hydrogel constructs, ranging from mold-based approaches to computer-aided biofabrication platforms, including extrusion-based bioprinting, light-assisted printing, and sacrificial manufacturing techniques [47]. Compared with extrusion-based bioprinting, which requires careful optimization of bioink rheology to ensure printability, structural fidelity, and cytocompatibility [9,48,49], RM can process precursor solutions spanning a broad viscosity range without relying on layer-by-layer deposition. Likewise, unlike sacrificial templating [50,51] and casting-based techniques [52,53], RM enables the direct, one-step fabrication of hollow constructs without the need for sacrificial materials, multiple processing steps, or post-fabrication assembly [30,47]. Conversely, computer-aided biofabrication techniques offer greater geometric freedom, facilitating the fabrication of highly branched and complex hollow architectures. Recent advances in stereolithography, melt electrowriting, and digital light processing have further expanded these capabilities, enabling the generation of increasingly sophisticated hollow constructs [[54], [55], [56]].

### 2.3 Cell viability and morphology in a protein-based tubular hydrogel

**Fig. 4:**
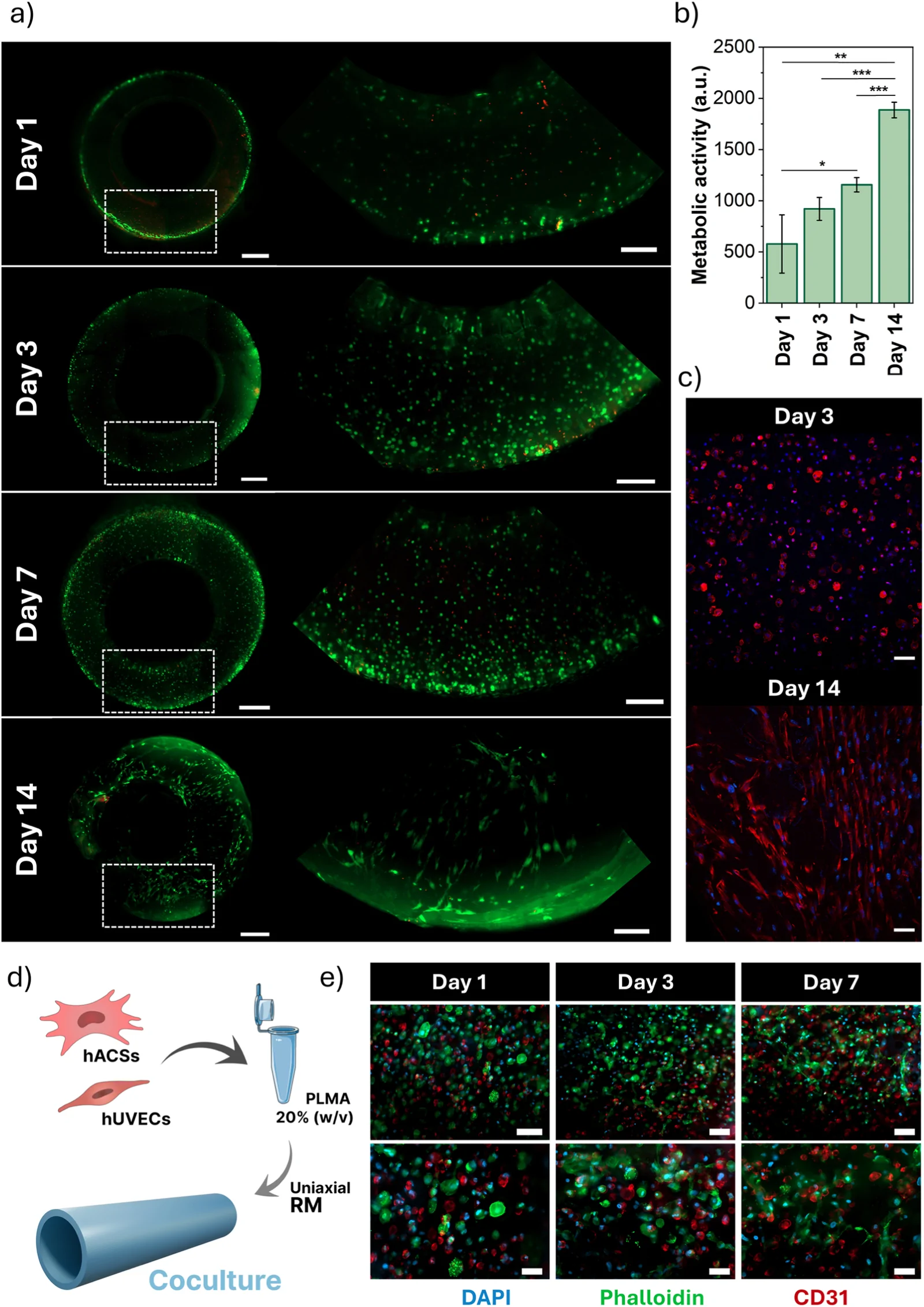
(a) Live/dead fluorescence microscopy images of hASCs encapsulated within the PLMA-based tubular template over 14 days, with Calcein AM (green) indicating live cells and propidium iodide (PI, red) indicating dead cells. The panel shows a full top view of the tubular template on the left (Scale bar = 500 μm) and a zoomed-in region on the right (Scale bar = 200 μm). (b) Quantitative analysis of cellular metabolic activity at the corresponding time points (*n* = 3). * *p*-value<0.05, ** *p*-value<0.01, *** p-value<0.001. (c) Fluorescence confocal microscopy images of cells within the PLMA-based hollow construct stained with DAPI (blue, nuclei) and phalloidin (red, F-actin cytoskeleton) after 3 and 14 days of incubation. Scale bar = 50 μm. (d) Scheme of the co-culture assay. hASCs and hUVECs were suspended in a 20% (w/v) PLMA solution, which was subsequently processed into hollow tubular constructs using uniaxial RM. (e) Immunofluorescence staining of CD31 (red) in hASCs/hUVECs co-cultures after 7 days of culture. Actin filaments and cell nuclei were counterstained with phalloidin (green) and DAPI (blue), respectively. Scale bars: 100 μm (top row) and 50 μm (bottom row).

As above-mentioned, hollow templates can be fabricated using PLMA, a pool of methacrylated human proteins that provides a rich niche of growth factors, cytokines, and other bioactive proteins [40]. With that in mind, human adipose-derived stem cells (hASCs) were loaded into a 20% (w/v) PLMA precursor solution containing 0.5% (w/v) of LAP photoinitiator at a density of 10 × 10^6^ cells/mL. Following photocrosslinking, the hollow constructs were incubated and cell viability and metabolic activity were evaluated at the desired timepoints (1, 3, 7, and 14 days). Live/dead fluorescence images (Fig. 4a) demonstrate that the cells remain largely viable throughout 14 days, with minimal signs of cell death. At early timepoints, fluorescence intensity profiles (Fig. S7) confirm that most cells are localized near the outer surface of the tubular construct, due to the centrifugal forces experienced during the RM process. By day 7, the profiles indicate a more homogeneous cell distribution across the scaffold wall, which becomes even more pronounced by day 14. These results quantitatively support the visual observations, demonstrating that the hollow architecture of the constructs facilitates nutrient and oxygen transport throughout the wall, enabling cell proliferation and progressive migration throughout the scaffold wall over time. In fact, by day 14, hASCs display a well-adherent morphology, with clear filopodial extensions indicating active cell–matrix interactions. To directly evaluate the effect of the tubular architecture on cell behavior, PLMA-based bulk hydrogels were fabricated using the same precursor volume and initial cell density as the hollow tubular constructs. Live/dead fluorescence images of the bulk hydrogels (Fig. S8) revealed cell viability comparable to that observed in the tubular constructs, particularly at the early timepoints. However, by days 7 and 14, a marked increase in the number of dead cells was observed. This behavior is likely associated with the hindered transport of oxygen and nutrients to encapsulated cells due to the bulk geometry of the hydrogel [57]. In contrast, the hollow tubular architecture reduces the diffusion distance across the scaffold wall, thereby facilitating mass transport and supporting sustained cell viability during long-term culture. Moreover, unlike the tubular constructs, cells within the bulk hydrogels remained predominantly rounded throughout the culture period with no evident signs of cell spreading. Metabolic activity of hASCs encapsulated within the hollow templates was also assessed over the course of 14 days (Fig. 4b).

A significant increase in metabolic activity was observed over time, indicating not only that the cells remained viable, as shown by the live/dead assay, but also that they were functionally active and capable of proliferation. Lastly, DAPI/phalloidin staining further corroborated the live/dead results, highlighting the progressive morphological adaptation of hASCs within the hollow templates. By day 3, cells appeared predominantly roundish, likely in response to the stress experienced during the RM process. By day 14, cells displayed a well-spread, adherent morphology with organized cytoskeletal structures, reflecting active cell–matrix interactions. Altogether, the data suggests that the architectural features imparted by RM influences hASCs organization, indicating that RM can provide a structural context compatible with long-term cellular maintenance. To further broaden the biological relevance of the platform, hASCs monocultures were extended to a hASCs/hUVECs co-culture model (Fig. 4d). Owing to the highly mechanosensitive nature of hUVECs [[58], [59], [60]], this model provides a more demanding assessment of the compatibility of the RM process. To this end, both cell types were encapsulated at a total cell density of 10 × 10^6^ cells/mL, at a 2:3 hASC:hUVEC ratio, within PLMA-based hollow constructs. Live/dead fluorescence imaging (Fig. S9) revealed high cell viability throughout the 7-day culture period, with negligible cell death, comparable to that observed for the hASC monocultures. However, as the live/dead assay does not distinguish between the two cell populations, immunofluorescence staining for CD31, a specific endothelial cell marker [61], was performed to identify hUVECs within the co-culture (Fig. 4e). Positive CD31 staining was observed throughout the 7-day culture period, confirming the presence and survival of hUVECs within the PLMA-based hollow constructs following the RM process. All together, these results demonstrate that the RM process is compatible with the encapsulation of different cell types, supporting its potential for multicellular biofabrication applications.

### 2.4 Biaxial rotational molding toward complex hollow templates

**Fig. 5:**
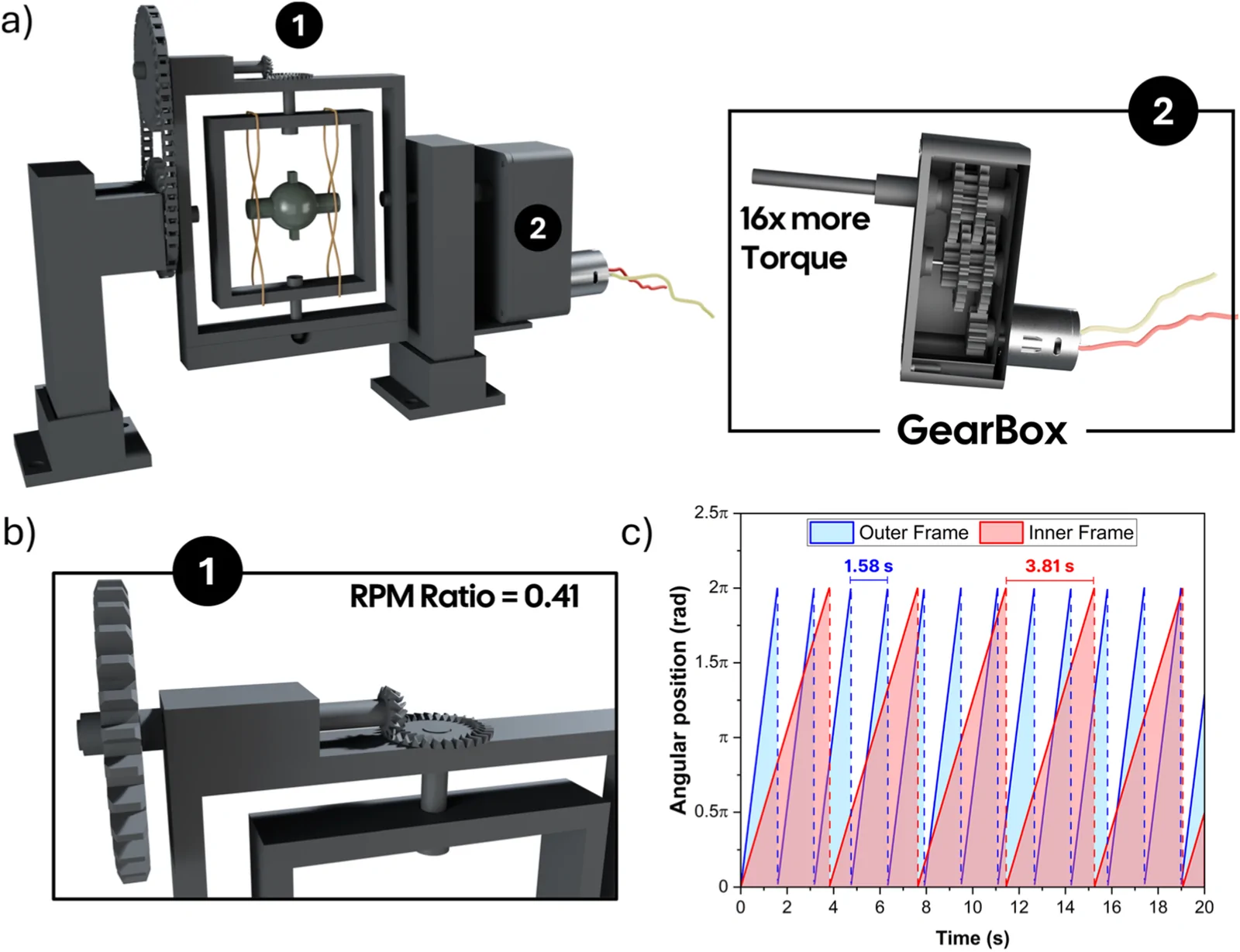
(a) Schematic 3D illustration of the biaxial RM apparatus, including the incorporated gearbox designed to increase the motor's torque for precise mold rotation. (b) Illustration of the gear-to-gear movement transfer, with a transmission ratio of 0.41. (c) Angular position θ (rad) of the outer (36 RPM) and inner (18 RPM) frames, measured at 1.3 V. The motion was modeled assuming constant angular velocity, neglecting energy losses and friction.

While uniaxial RM offers a first step toward mimicking the ubiquitous tubular geometries in the human body [62,63], many physiological structures exhibit curvatures, enclosed volumes, or complex three-dimensional shapes that remain challenging to reproduce with conventional fabrication methods [[64], [65], [66]]. Having demonstrated the feasibility of generating well-defined hollow tubular geometries via uniaxial RM, we next explored whether biaxial rotation could extend this approach to more intricate morphologies. This capability mirrors the versatility of RM in the world of thermoplastics but remains unexplored in the context of soft biomaterials and hydrogels, potentially offering a new biofabrication strategy for complex hollow structures. To harness this potential, we designed a custom apparatus drawing on the principles of traditional RM machines. The 3D-printed biaxial RM prototype is shown in Fig. 5a and Fig. S10.

To ensure consistent rotation, the motor output is transmitted to the outer frame through a gearbox (Fig. S11), which increases the torque by 16-fold, thereby enabling a stable transfer of motion. To achieve biaxial rotation, the inner frame needs to rotate around a second axis that is orthogonal to the outer frame's axis of rotation. This is accomplished by transferring motion from the outer frame through a gear-and-gear system incorporating a ratio of *ca.* 0.42 (Fig. 5b), which sets the rotational speed of the inner frame. In traditional RM, an integer ratio of 4:1 is known to provide uniform material distribution, however, non-integer ratios are preferred to avoid repeated patterned artifacts [67]. Our biaxial RM prototype implements a non-integer speed ratio of ∼2.4:1 (outer frame:inner frame), achieving similar benefits for the distribution of the precursor mixture. Additionally, the rotational speeds of both frames were measured and converted to angular velocity (ω). The angular position (θ) as a function of time (t) can be expressed as: θ(t)=ωt. As shown in Fig. 5c and Movie S2, the outer frame completes a full rotation in 1.58 s, while the inner frame completes one in 3.81 s, resulting in an experimental speed ratio of about 0.41. This close agreement between the measured speed ratio and the designed gear ratio indicates negligible transmission losses, confirming efficient motion transfer from the outer to the inner frame.

**Fig. 6:**
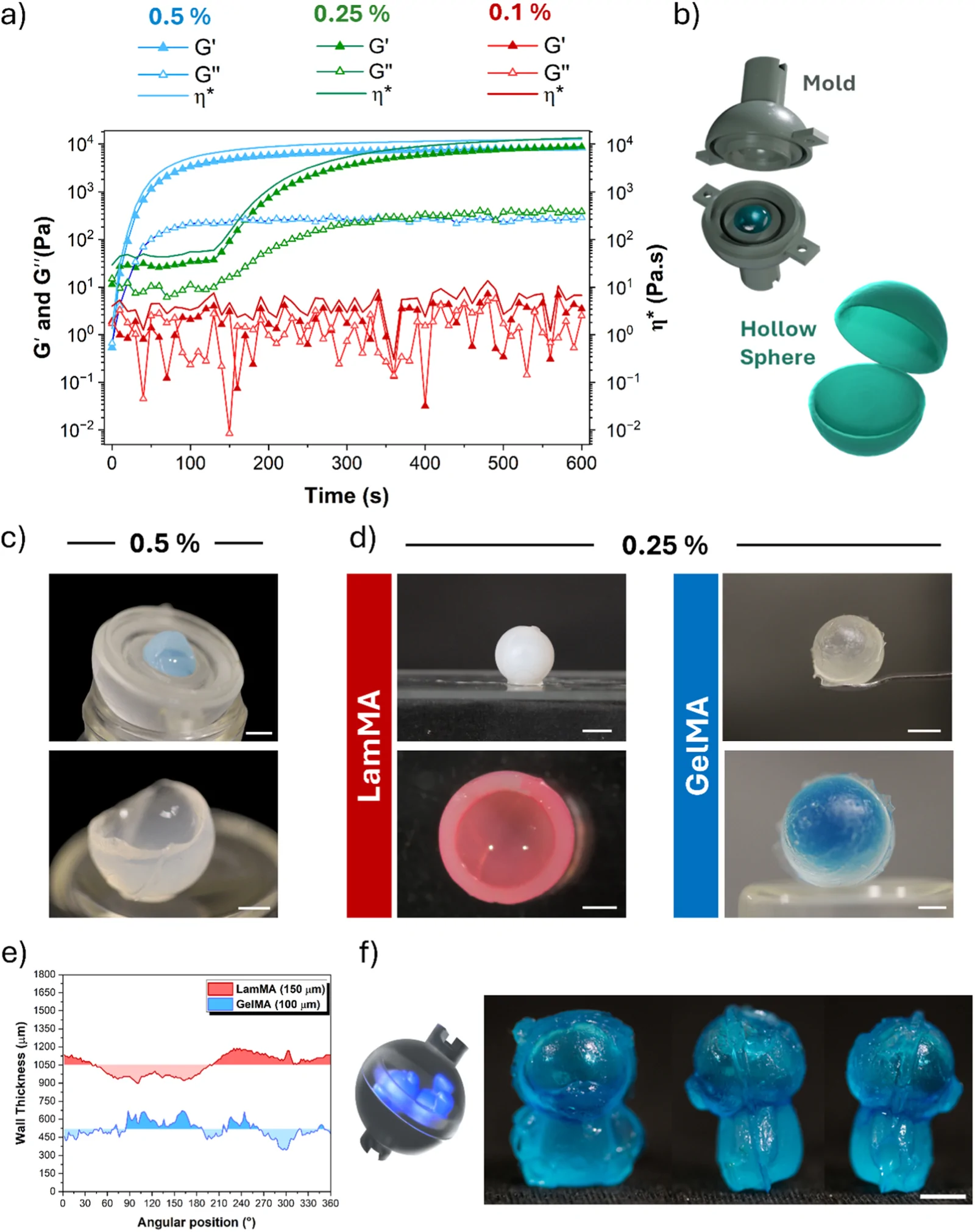
(a) Rheological time-sweep measurements of LamMA 20% (w/v) with increasing APS/TEMED concentrations, acquired at 25 °C. (b) Schematic 3D view of the 3D-printed mold used for the fabrication of hollow spheres. (c) Digital images of an incomplete hollow sphere fabricated with LamMA 20% (w/v) and 0.5% (w/v) APS/TEMED; the blue color was digitally added for visualization. Top image scale bar = 4 mm; bottom image scale bar = 2 mm. (d) Digital images of well-defined hollow spheres fabricated either with LamMA 20% (w/v) and 0.25% (w/v) APS/TEMED or with 15% (w/v) GelMA and 0.5% (w/v) LAP. Top images scale bar = 4 mm; bottom images scale bar = 2 mm. (e) Wall thickness variation of a hollow spheres fabricated with LamMA and GelMA. Measurements were acquired at 2° angular increments. Values above and below the calculated average are highlighted in dark and light colors, respectively. (f) 3D view of a complex structure mold and corresponding digital images obtained from biaxial RM using 550 μL of GelMA with 0.5% (w/v) LAP. Scale bar = 4 mm. All measurements were performed using ImageJ software.

Unlike uniaxial rotation, where the fast gelation kinetics promoted by the photocrosslinking step can be used for fixing the tubular shape, biaxial RM, which is not dependent on centrifugal forces, presents a different scenario, where slower gelation kinetics are required to ensure proper coverage of the inner surface of the mold. For instance, in traditional RM, the cooling cycle is longer than the heating cycle to allow thermoplastic annealing to proceed at a controlled rate, ensuring homogeneous curing throughout the mold [68,69]. As such, photopolymerization may not be suitable for biaxial RM. Alternatively, redox-initiated radical polymerization can provide an additional level of control over gelation kinetics. Ammonium persulfate (APS) and tetramethylethylenediamine (TEMED) are widely used redox pairs [[70], [71], [72]] that enable controlled modulation over the crosslinking rate by adjusting the concentration of the oxidizing and reducing agent [73]. Based on these considerations, increased concentrations of APS and TEMED (1:1 eq.) were mixed with a precursor solution of LamMA 20% (w/v), and their gelation profile was analyzed through rheological time-sweep measurements. As shown in Fig. 6a, a concentration of 0.1% (w/v) APS/TEMED did not induce gelation, as reflected by the flat/lower storage (G′) and loss (G″) modulus profiles, in contrast, concentrations of 0.25% and 0.5% (w/v) produced a pronounced increase in both moduli. Notably, the highest APS/TEMED concentration triggered an abrupt onset of gelation, characterized by a sharp increase in G′ and G″, suggesting instantaneous gelation that may compromise the uniform coating of the mold resulting in incomplete molding. The complex viscosity (η*) exhibited a similar trend, with an immediate increase, further confirming the fast gelation. At 0.25% (w/v) an induction period of approximately 100 s was observed, during which G′, G″, and η* remained essentially flat, followed by an increase to values comparable to those obtained with 0.5% (w/v), reflecting a delayed yet complete gelation. Such delayed gelation may be advantageous in the context of RM, as it provides a sufficient temporal window to load the precursor solution into the mold and initiate biaxial rotation, allowing the solution to uniformly coat the mold before crosslinking is complete. Moreover, the rate at which G′ increases after the onset can also affect the uniformity of mold coverage, as a slower gelation rate allows the precursor mixture to evenly coat the mold during the RM process. To quantitatively compare the gelation rate between the two formulations, the G′ curves were fitted using a Boltzmann sigmoidal model (Equation (1)) as displayed in Fig. S12.

Eq (1) $$y=A_{2}+\frac{A_{1}-A_{2}}{1+e^{\left(\frac{x-x_{0}}{dx}\right)}}$$

The maximum gelation rate (maximum slope) occurs at the inflection point of the curve (${x=x}_{0}$) [74], at that point the gelation rate is given by: ${\frac{dy}{dx}\;|}_{x=x_{0}}=\left|\frac{A_{1}-A_{2}}{4dx}\right|$. Based on the fitting parameters obtained for both formulations (Table S1), the maximum slopes of the G′ curves were *ca.* 63 and 30 Pa s^−1^ for the 0.5% and 0.25% (w/v) APS/TEMED formulations, respectively. This confirms that the higher APS/TEMED concentration induces a much faster gelation rate, whereas the slower gelation observed for the 0.25% formulation favors a more uniform coating of the mold during the sol-to-gel transition, supporting its suitability for RM. Overall, tuning the APS/TEMED concentration provides a straightforward strategy to control both the onset and kinetics of gelation, enabling tailored sol-to-gel transitions suitable for RM.

To validate these findings in a experimental setting, the gelation behavior of LamMA with 0.25% and 0.5% (w/v) APS/TEMED was further evaluated using our biaxial RM prototype. A spherical mold (Ø 8 mm) was selected due to the reproducibility of a hollow sphere, facilitating the assessment of structural homogeneity and enabling straightforward evaluation of material distribution throughout the mold (Fig. 6b). Consistent with the rheological data, RM of the precursor solution at the highest APS/TEMED concentration resulted in incomplete gelation, yielding a hollow sphere that was only partially formed as displayed in Fig. 6c. In contrast, a fully formed, well-defined hollow sphere was obtained with 0.25% (w/v) APS/TEMED, exhibiting a uniform material distribution across the surface (Fig. 6d). Notably, by exploiting the upper critical solution temperature (UCST) of GelMA [75], well-defined hollow spheres can be generated without the need for redox initiators. The GelMA precursor (15% (w/v)) solution containing LAP (0.5% (w/v)) was heated to 37 °C, loaded into the mold, and placed under biaxial rotation. While cooling to RT, gelation takes place, allowing the mold to be fully coated. The pre-formed hollow sphere was then exposed to blue light, triggering photocrosslinking to preserve the spherical geometry. The biaxial RM procedure yielded well-defined protein-based hollow spheres, as presented in Fig. 6d. Furthermore, injection of blue dye into the GelMA sphere confirmed the presence of a hollow core, clearly demonstrating the successful fabrication of complex 3D hollow architectures. Owing to their uniform spherical geometry, the wall thickness (μm) the sphere can be estimated according to the following relationship: Wallthickness(v)=4−(268−v43·π)13, where 268 (mm^3^) and 4 (mm) is the total volume and radius of the spherical mold, respectively, and v (μL) describes the loaded volume of the precursor mixture. Based on this relationship, we next evaluated how closely the experimental structures matched these predictions using a hollow sphere produced from 150 μL of LamMA 20% (w/v) with 0.25% (w/v) APS/TEMED and from 100 μL of GelMA 15% (w/v) and 0.5% (w/v) LAP. Based on the loaded volume, the calculated wall thickness was 576 and 957 μm for 100 and 150 μL, respectively. As shown in Fig. S13, the measured values (1051.9 ± 82.3 μm for the LamMA construct and 518.3 ± 69.3 μm for the GelMA construct) were consistent with the predicted values (black dash lines). Furthermore, as shown in Fig. 6e, the wall thickness remained relatively constant along the entire sphere, with only small deviations from the average values. Together, these results highlight the ability of biaxial RM to generate programmable and uniform hollow spheres. Although the hollow sphere serves as a useful benchmark, its simplicity does not fully challenge the capabilities of the proposed RM technique. To demonstrate that the proposed method can faithfully replicate more demanding geometries, we subsequently tested a mold incorporating pronounced curvatures and multiple angular transitions. This mold, serving as the negative template for the RM process, was 3D printed in the shape of a gummy bear. A precursor mixture (550 μL) of GelMA 15% (w/v) and LAP 0.5% (w/v) was heated to 37 °C, loaded into the mold, and immediately subjected to biaxial rotation for 15-20 min. After the RM process, the resulting GelMA structure was crosslinked under blue light. Fig. 6f shows the resulting 3D construct alongside the mold used. Remarkably, most of the intricate details of the model were captured using RM, yielding a construct that closely matches its original design. Even at a reduced scale (∼15 mm height), RM successfully preserved the key geometrical features of the design, highlighting the method's ability to replicate fine details with high fidelity. Although the level of detail obtained here can be compared to what can be achieved with high-resolution volumetric 3D printing using the same protein-based material [76], this construct embodies a geometry that remains particularly challenging to obtain through conventional extrusion-based 3D bioprinting methods.

## 3 Conclusion

In conclusion, we demonstrate for the first time that RM, a technique traditionally used in industrial polymer processing, can be adapted into a cost-effective, versatile and high programable biofabrication platform for the generation of hollow templates. Both uniaxial and biaxial RM provide predictable control over geometry, wall thickness, and topology, while maintaining compatibility with a broad range of methacrylated polymers crosslinked using distinct chemical routes. Extending this approach, multilayered biomaterial constructs can be fabricated with diverse polymeric compositions, allowing the creation of complex, hierarchically organized hollow structures with tunable interfaces. This platform not only allows the incorporation of different polymer layers and cell-laden formulations, but also ensures reproducible structural fidelity across scales, establishing a robust route for engineering intricate hollow architectures. Looking forward, RM-based biofabrication offers an accessible route to engineer complex hollow constructs for tissue models, biohybrid living actuators, and organ-mimetic systems, opening new avenues for regenerative medicine, synthetic tissue design, and the development of next-generation biomaterials.

## 4 Materials and methods

### 4.1 Materials

Laminarin (from *Eisenia bicyclis*) and lithium phenyl-2,4,6-trimethylbenzoylphosphinate (LAP) were purchased from Biosynth. Porcine skin gelatin (gel strength 300, type A), low viscosity sodium alginate (from brown algae), calcium chloride anhydrous, ethylenediaminetetraacetic acid (EDTA), and 6-aminofluorescein were acquired from Sigma-Aldrich. Glycidyl methacrylate (stabilized with MEHQ, 95%), methacrylic anhydride (>97%), and deuterium oxide (99.8%) were purchased from TCI Chemicals. 4-Dimethylaminopyridine (DMAP, 99%) was obtained from ACROS Organics. Metacrylated platelet lysates (PLMA) were bought from Metatissue. The DC-DC converter module (LM2596) and the DC motor were purchased from Mauser. Unless otherwise specified, all chemicals were used as received without further purification.

### 4.2 3D printing parts

All CAD files were designed using Sharp3D (version 5.991.9804.0), processed using FlashPrint 5, and printed on a Flashforge Creator 3 V2 using PLA filament (The Filament by Spectrum) with a 0.4 mm nozzle. Molds for the biaxial RM experiments were fabricated using an Anycubic Photon M3 Plus SLA printer (Anycubic, Shenzhen, China) equipped with a 6k monochrome LCD, with a resolution of 34 μm, and printed with Anycubic Craftsman resin. Printed molds were rinsed in isopropanol to remove residual uncured resin, dried, and post-cured under ultraviolet light for 30 min.

#### 4.2.1 Uniaxial RM apparatus

The single-axis apparatus was constructed on a custom 3D-printed frame, with an ON/OFF switch regulating a DC-DC converter module (LM2596) that powered the DC motor, to which a 3D-printed holder could be attached depending on the glass mold used.

#### 4.2.2 Biaxial RM apparatus

The same electronic configuration as described for the uniaxial system was employed to drive the motors in this setup. An additional gearbox was incorporated between the motor and the outer frame to increase torque, ensuring reliable operation of the system under load.

### 4.3 Synthesis of the methacrylated polymers

#### 4.3.1 Methacrylated laminarin (LamMA)

Methacrylated laminarin (LamMA) was synthesized as previously reported by our group [38]. Laminarin (1.0 g, 5.56 mmol of glucose units) was dissolved in 10.0 mL of dry DMSO under a N_2_ atmosphere. To this mixture, 167.0 mg of 4-(Dimethylamino)pyridine (DMAP, 1.36 mmol) was added, followed by 56.9 mg (0.4 mmol) of glycidyl methacrylate. The reaction was allowed to proceed at room temperature (RT) for 48 h and was stopped by the addition of an equimolar amount of 37% (v/v) HCl. The resulting product was purified by dialysis against distilled water using a 3.5 kDa molecular weight cut-off (MWCO) membrane. Finally, LamMA was recovered by freeze-drying as a white foam and store protected from the light. The success of the reaction was confirmed by ^1^H NMR spectroscopy (Fig. S14a), and the degree of substitutions (DS) was determined according to previously reported methods [38].

#### 4.3.2 Methacrylated gelatin (GelMA)

Methacrylated gelatin (GelMA) was synthesized as reported elsewhere [46]. Briefly, Gelatin (2.0 g) was dissolved in 20.0 mL of PBS and heated to 50 °C. Methacrylic anhydride (800 μL) was added dropwise to the solution and stirred at 50 °C for 4 h. After which an equimolar amount of NaOH (1M) was added to stop the reaction. The reaction mixture was then purified by dialysis against distilled water using a 3.5 kDa MWCO membrane for at least 3 days, with the water changed twice daily. Purified GelMA was recovered by freeze-drying as a white foam and stored at 4 °C protected from light. The success of the reaction was confirmed by ^1^H NMR spectroscopy (Fig. S14b), and the DS was determined according to previously reported methods [46,77].

### 4.4 Fabrication of the hollow tubular templates

For the fabrication of the hollow templates, the corresponding methacrylated polymer (LamMA, GelMA, or PLMA) was dissolved in phosphate buffered saline (PBS, pH 7.4) at the desired concentration (20% w/v for LamMA and PLMA, and 15% w/v for GelMA) together with 0.5% w/v LAP. After complete dissolution, the precursor solution was loaded into the mold and secured within the rotational apparatus. Uniaxial rotation was initiated (1750 rpm unless otherwise stated), and once a steady rotational regime was reached (<5 s), where centrifugal forces became sufficient to displace the material outward and form a central lumen, the constructs were photocrosslinked under UV/blue light (λ = 385–515 nm; Valo Grand; 3 min, 1000 mW/cm^2^). Prior to any experiments, the glass molds were treated with a water-repellent solution, allowed to dry at RT, and rinsed with PBS before use.

### 4.5 Fabrication of the bilayer tubular templates

The fabrication of the bilayer constructs followed a procedure analogous to that described above. After forming and photocrosslinking the first layer, the second precursor mixture was introduced into the hollow lumen of the first layer. Rotation was then reinitiated to ensure uniform coating of the inner surface, after which the second layer was photocrosslinked using the same irradiation parameters.

#### 4.5.1 Fluorescence microscopy

The bilayer construct, composed of an outer LamMA layer and an inner GelMA layer, was incubated for 10 min in a 5.0 mM 6-aminofluorescein solution and subsequently washed with PBS. Fluorescence images were then acquired using a confocal laser scanning microscope (CLSM 900, Carl Zeiss Microscopy, Germany) equipped with a 10× objective.

#### 4.5.2 Scanning electron microscopy (SEM)

SEM was employed to examine the morphology of the bilayer tubular construct. Thin cross-sections were obtained with a scalpel, mounted onto metal stubs containing O.C.T. compound (Tissue-Tek, Sakura, Japan), and imaged using an SU-3800 system (Hitachi, Japan) operated at −25 °C. The system was equipped with a standard cooling stage (Deben, UK) with a temperature range of −25 °C to 50 °C.

### 4.6 Introducing porosity into the wall

To customize the wall's topology , alginate-Ca^2+^ beads were mixed with the precursor mixture (LamMA 20% (w/v) and 0.5% (w/v) LAP) and subjected to RM. After the photocrosslinking step the tubular constructs were treated with EDTA (0.02 M, pH 6.7) for 5 min at RT to liquify the alginate-Ca^2+^ beads. The alginate-Ca^2+^ beads were fabricated by electrohydrodynamic atomization (EHDA) as previously reported [78]. Briefly, alginate (2.5% w/v) was dissolved in 25 mM MES (pH 6.7) containing NaCl (0.15 M) under gentle stirring. The solution was extruded by EHDA using a 22 G needle, an 8 cm tip-to-collector distance, a 50 mL h^−1^ flow rate, and an applied voltage of 7 kV. Droplets were collected directly into a 0.1 M CaCl_2_ bath (pH 6.7) under mild agitation at RT for 20 min, after which the resulting beads were recovered using a microsieve.

### 4.7 Cell culture and biological performance

To evaluate the *in vitro* biological performance, human adipose-derived mesenchymal stem cells (hASCs) (ATCC® PCS-500-011™) were used as an adherent model cell type. Cells were expanded in T-175 flasks using Minimum Essential Medium Alpha (α-MEM, Sigma-Aldrich) supplemented with 10% (v/v) FBS and 1% (v/v) antibiotic/antimycotic under standard culture conditions. Once confluent, hASCs were detached with trypsin–EDTA and resuspended at a density of 10 × 10^6^ cells/mL in an aqueous precursor solution containing 20% (w/v) PLMA and 0.5% (w/v) LAP. The resulting cell-laden mixture was then processed by uniaxial RM as described above. Human umbilical vein endothelial cells (hUVECs) were obtained under a cooperation agreement between the COMPASS Research Group situated in CICECO – Aveiro Institute of Materials, University of Aveiro, the Centro Hospitalar do Baixo Vouga (Aveiro, Portugal) and Hospital da Luz (Aveiro, Portugal), after approval of the Competent Ethics Committee (CEC, agreement 10052020) with informed consent from all parties involved. Following collection, umbilical cords were transported at 4 °C in DPBS supplemented with 1% (v/v) penicillin–streptomycin (PenStrep, Thermo Fisher Scientific). For the isolation of HUVECs, the umbilical cord was washed in PBS and blood clots were removed before enzymatic digestion using 0.1% (w/v) collagenase type IA (MP Biomedicals, USA). The isolated hUVECs were expanded in tissue culture flasks coated with 0.7% (w/v) gelatin and maintained at 37 °C in a humidified atmosphere containing 5% CO_2_. Cells were cultured in M199 medium (Sigma-Aldrich) supplemented with 20% (v/v) fetal bovine serum (FBS), 10% (v/v) heparin (100 mg/mL, Sigma-Aldrich), 1% (v/v) endothelial cell growth supplement (ECGS, 40 mg/mL, Merck, Germany), and 1% (v/v) antibiotic/antimycotic.

#### 4.7.1 Cell viability assays

Cell-laden PLMA-based tubular scaffolds were maintained in culture for up to 14 days, and cell viability was evaluated at predetermined time points (1, 3, 7, and 14 days) using a Live/Dead assay. At each timepoint, constructs were incubated for 20 min at 37 °C in Dulbecco's Phosphate Buffered Saline (DPBS, Sigma-Aldrich) containing calcein-AM (2 μg/mL, ThermoFisher Scientific) and propidium iodide (PI, 1 μg/mL, ThermoFisher Scientific), protected from light. Fluorescence images were acquired using a Zeiss Imager M2 widefield microscope equipped with a 3-megapixel camera (Carl Zeiss Microscopy, Oberkochen, Germany), and image analysis was performed with ZEN v2.3 Blue Edition software (Zeiss). For the co-culture assays, hASCs and hUVECs were mixed at a 2:3 ratio and a total cell density of 10 × 10^6^ cells/mL before encapsulation in 20% (w/v) PLMA precursor solution and processed by uniaxial RM. The cell-laden constructs were maintained for 7 days in culture medium composed of (2:3) α-MEM (supplemented with 10% (v/v) of FBS and 1% (v/v) antibiotic/antimycotic.) and M199 (supplemented with 1% (v/v) of ECGS, 10% (v/v) of heparin, 20% (v/v) FBS, and 1% (v/v) antibiotic/antimycotic). Cell viability was subsequently evaluated (1, 3, and 7 days) following the same Live/Dead protocol described above.

#### 4.7.2 DAPI/phalloidin staining

To analyze cell morphology, cells were stained with phalloidin (1:40 (v/v) in DPBS, Flash Phalloidin™ Red 594, BioLegend) at RT for 20 min, followed by incubation with DAPI (1:1000 (v/v) in DPBS, Thermo Fisher Scientific), for 5 min at RT, protected from light. For the immunofluorescence staining, samples were permeabilized with 0.1% (v/v) Triton X (Merck) for 10 min, treated with 1% (w/v) BSA (Bovine serum albumin, Sigma-Aldrich) for 1 h, and incubated overnight at 4 °C with a mouse anti-CD31 primary antibody (1:50 in 1% BSA, Dako). After washing, samples were incubated with an Alexa Fluor 647 goat anti-mouse secondary antibody (1:100 in HBSS, ThermoFisher Scientific), for 1 h at RT, followed by phalloidin and DAPI staining as described above. Stained samples were imaged using a Zeiss Imager M2 widefield fluorescence microscope equipped with a 3-megapixel camera (Carl Zeiss Microscopy, Oberkochen, Germany). Image analysis was performed using ZEN v2.3 Blue Edition software (Zeiss).

#### 4.7.3 Metabolic activity

Metabolic activity of hASCs encapsulated within the PLMA-based tubular hydrogels was quantified using the AlamarBlue Cell Viability Reagent (Alfagene, Lisbon, Portugal). After removing the culture medium, a 10% (v/v) AlamarBlue solution prepared in α-MEM was added to each sample and incubated for 4 h at 37 °C. Fluorescence was then measured at the predetermined time points using a Synergy HTX multi-mode microplate reader (BioTek Instruments, Winooski, VT) at excitation/emission wavelengths of 570/585 nm.

### 4.8 Rheological measurements

Time-sweep measurements were performed to assess the gelation kinetics of LamMA (20% w/v) in the presence of APS and TEMED at different concentrations. Experiments were carried out on a Kinexus Lab + rotational rheometer (Malvern PANalytical, U.K.) equipped with an 8 mm parallel plate geometry, with the gap set to 0.25 mm. Oscillatory measurements were conducted at a fixed strain of 0.1% and a frequency of 0.1 Hz at 25 °C throughout the duration of the time sweep. Steady shear measurements were additionally performed to determine the viscosity of the precursor solutions. Viscosity was recorded as a function of applied shear stress using a stainless steel cone-plate geometry, with cone diameter 25 mm and 1° angle. The viscosity values correspond to the Newtonian plateau observed at low shear stresses.

### 4.9 Fabrication of complex geometries using biaxial RM apparatus

#### 4.9.1 LamMA hollow templates

Firstly, stock solutions of APS and TEMED (10% w/v) were prepared separately and stored at 4 °C. The calculated amount of APS was first mixed with a 20% (w/v) LamMA solution and homogenized using a vortex, followed by the addition of the corresponding volume of TEMED. Immediately after mixing, the precursor solution was transferred into the mold, and biaxial rotation (outer frame: 36 rpm; inner frame: 18 rpm) was initiated for 10–15 min. Finally, the mold was disassembled, and the hollow template was carefully retrieved.

#### 4.9.2 GelMA hollow templates

A 15% (w/v) GelMA solution was prepared in PBS containing 0.5% (w/v) LAP and stirred at 37 °C on a thermomixer. Prior to loading, the mold was equilibrated at 37 °C. The precursor solution was then introduced into the mold, and biaxial rotation (outer frame: 36 rpm; inner frame: 18 rpm) was performed for 15–20 min. Finally, the mold was disassembled, and the hollow template was carefully retrieved.

### 4.10 Statistical analysis

All data were statistically evaluated using GraphPad Prism software (version 10.1.0) and are reported as mean ± standard deviation (*n* = 3). Statistical differences were considered significant when *p*-value < 0.05, as determined by an unpaired *t*-test, unless otherwise specified.

## CRediT authorship contribution statement

**Luís P.G. Monteiro:** Writing – original draft, Methodology, Investigation, Formal analysis, Data curation. **João M.M. Rodrigues:** Writing – review & editing, Supervision, Methodology, Funding acquisition, Conceptualization. **João F. Mano:** Writing – review & editing, Validation, Supervision, Resources, Project administration, Funding acquisition, Conceptualization.

## Declaration of competing interest

The authors declare that they have no known competing financial interests or personal relationships that could have appeared to influence the work reported in this paper.

## Data Availability

Data will be made available on request.
