# Supplementary material for: A one-step volumetric biofabrication platform for complex hydrogel-based hollow biomaterials
Source: Mater Today Bio. 2026 Aug 28;40:103594. doi: 10.1016/j.mtbio.2026.103594 (PMC13571706; doi:10.1016/j.mtbio.2026.103594)
Supplement: Supplementary file 1 [file mmc1.pdf]

SUPPORTING INFORMATION

A One-Step Volumetric Biofabrication Platform for Complex Hydrogel-Based Hollow Biomaterials

Luís P. G. Monteiro, João M. M. Rodrigues*, João F. Mano*

CICECO – Aveiro Institute of Materials, Department of Chemistry, University of Aveiro, 3810-193 Aveiro, Portugal

Corresponding author: jrodrigues@ua.pt; jmano@ua.pt

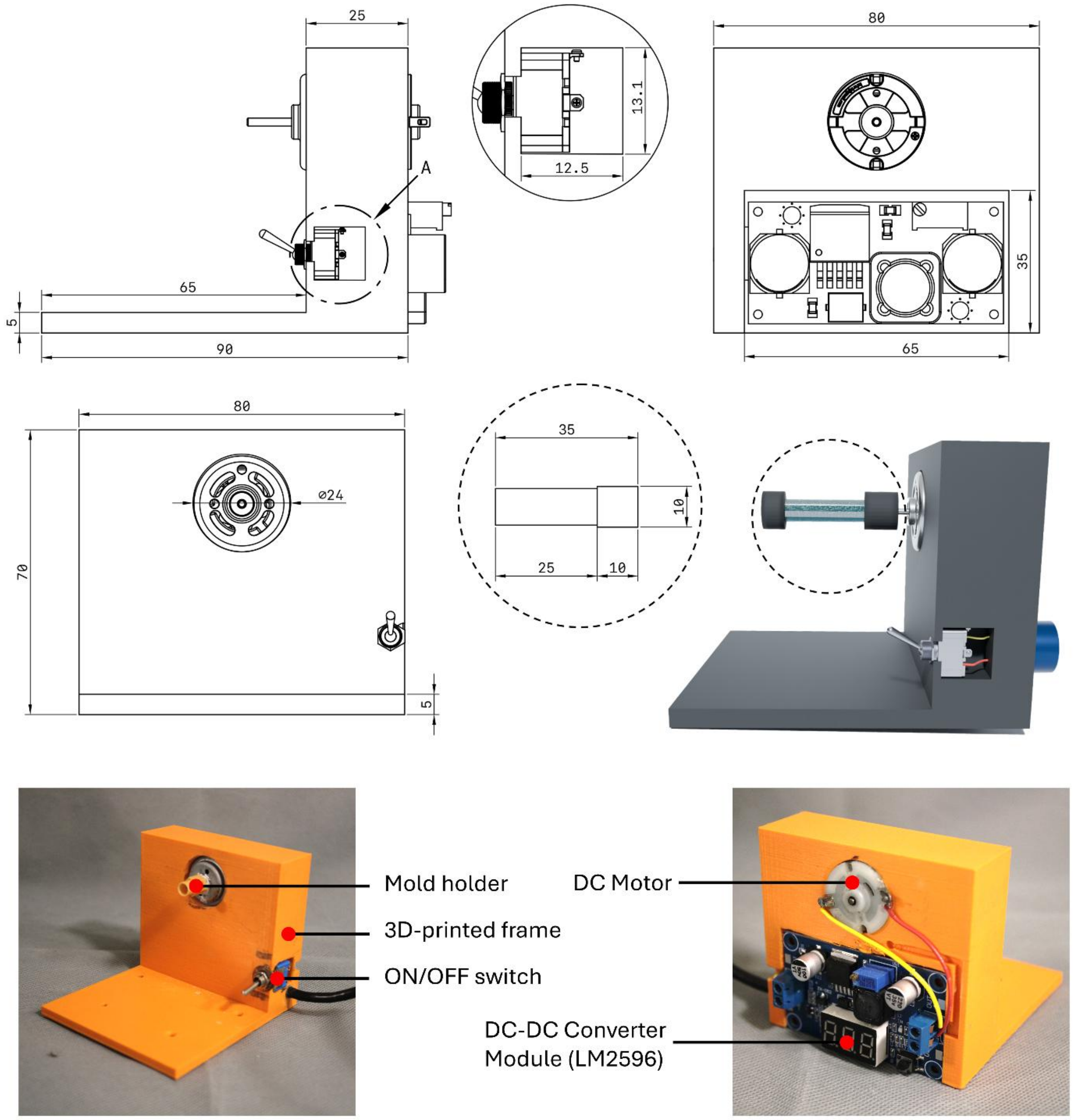

Figure S1: Multi-view technical drawings of the uniaxial RM apparatus, including all dimensions, alongside labeled digital images of the designed components.

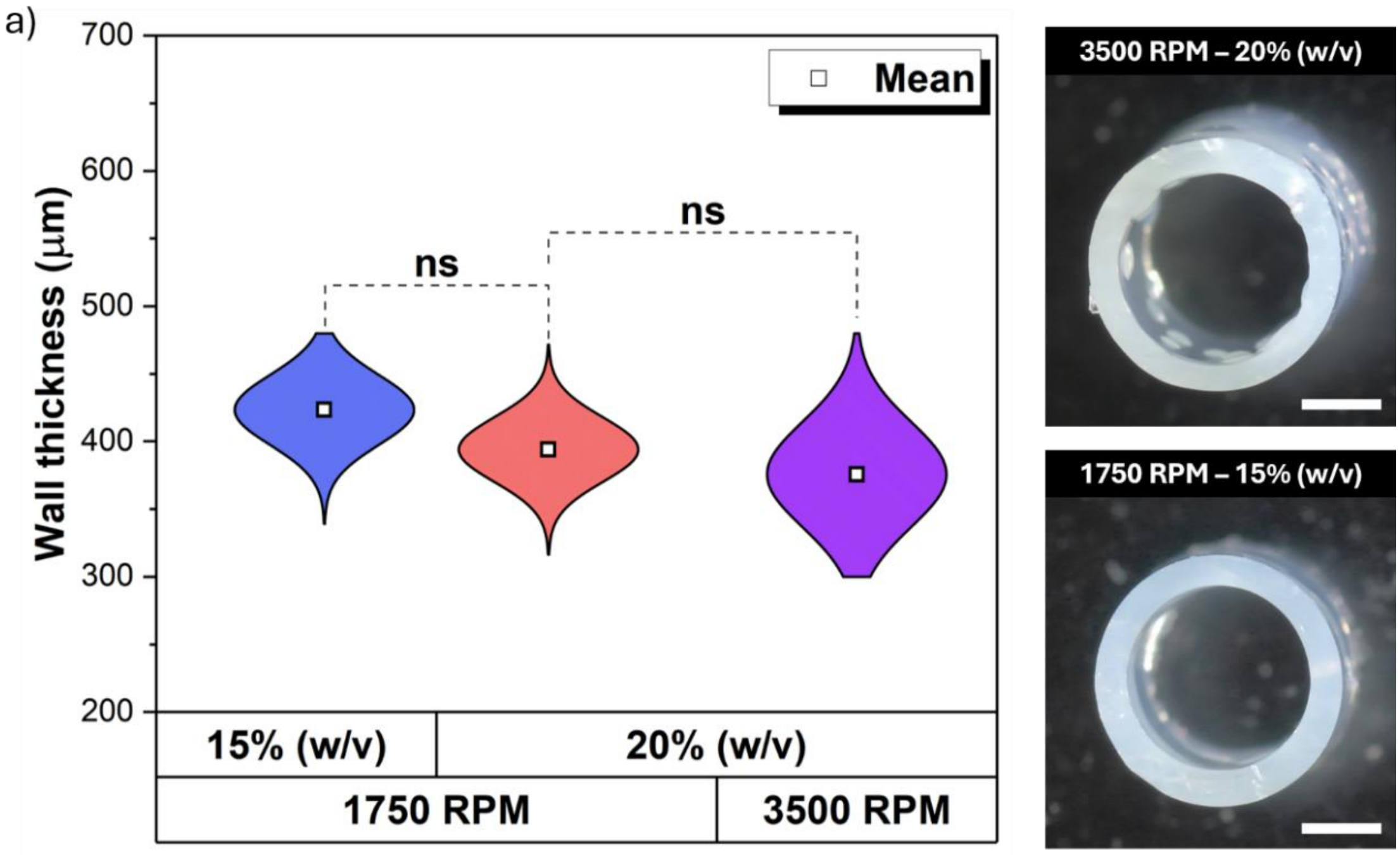

Figure S2: Wall thickness of tubular templates fabricated in a 1.7 mm radius glass mold using 100 µL of LamMA precursor solution as a function of rotation speed and polymer concentration. Measurements were acquired at 2° angular increments around the entire tube circumference. Scale bar = 1 mm.

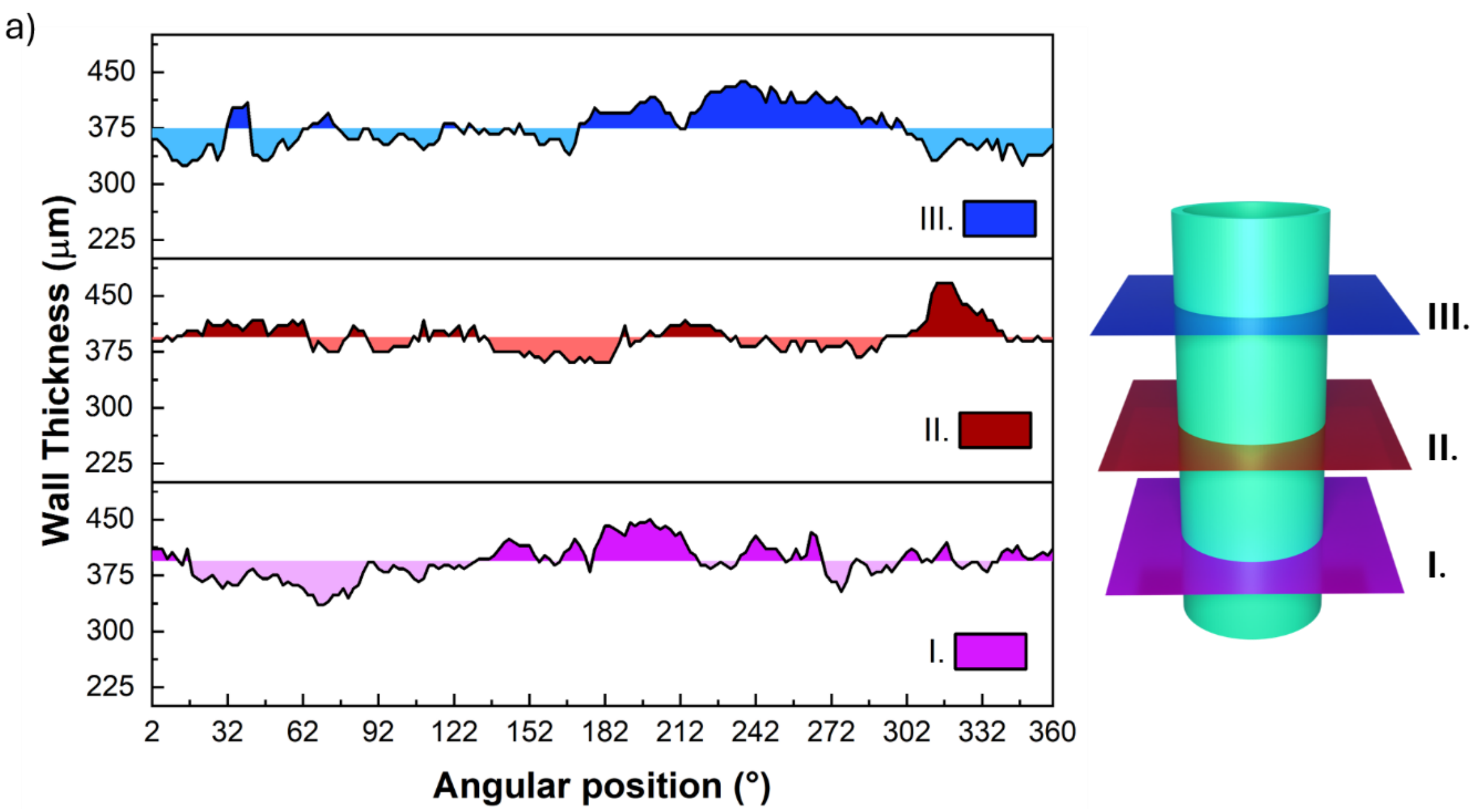

Figure S3: Wall thickness variation at three cross-sectional positions of a tubular template fabricated in a glass mold with an inner radius of 1.7 mm using 100 µL of 20% (w/v) LamMA precursor solution. Measurements were acquired at 2° angular increments around the entire tube circumference. Values above and below the calculated average are highlighted in dark and light colors, respectively.

a) b)

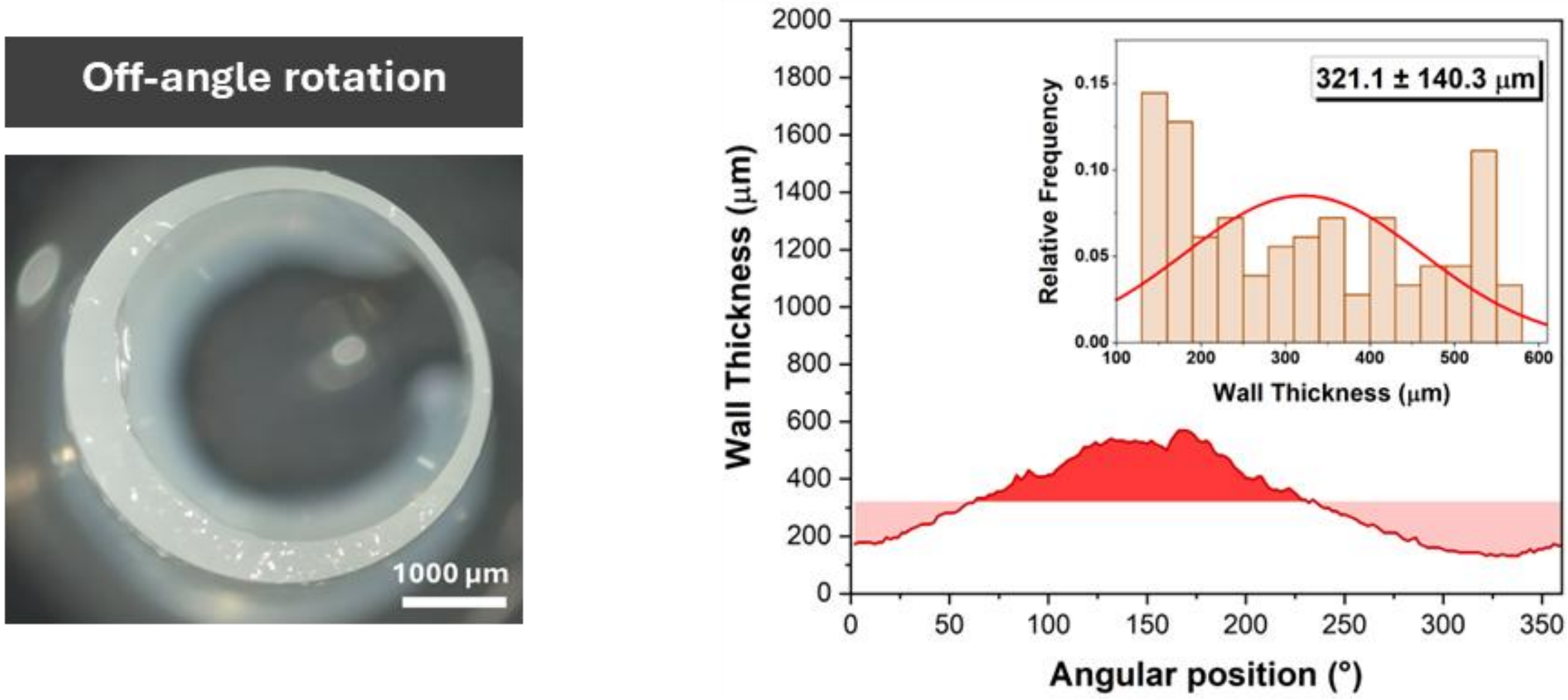

Figure S4: (a) Digital image of a LamMA hollow template (100 µL) produced by off-axis rotation. Scale bar = 1000 µm. (b) Corresponding wall thickness variation the tubular template fabricated using 100 µL of 20% (w/v) LamMA. Measurements were acquired at 2° angular increments. Values above and below the calculated average are highlighted in dark and light colors, respectively. The inset shows the corresponding histogram and fitted normal distribution curve (red line). Thickness measurements revealed that the maximum and minimum wall thickness values deviated from the mean thickness by 77.2 % and 58.8 %, respectively.

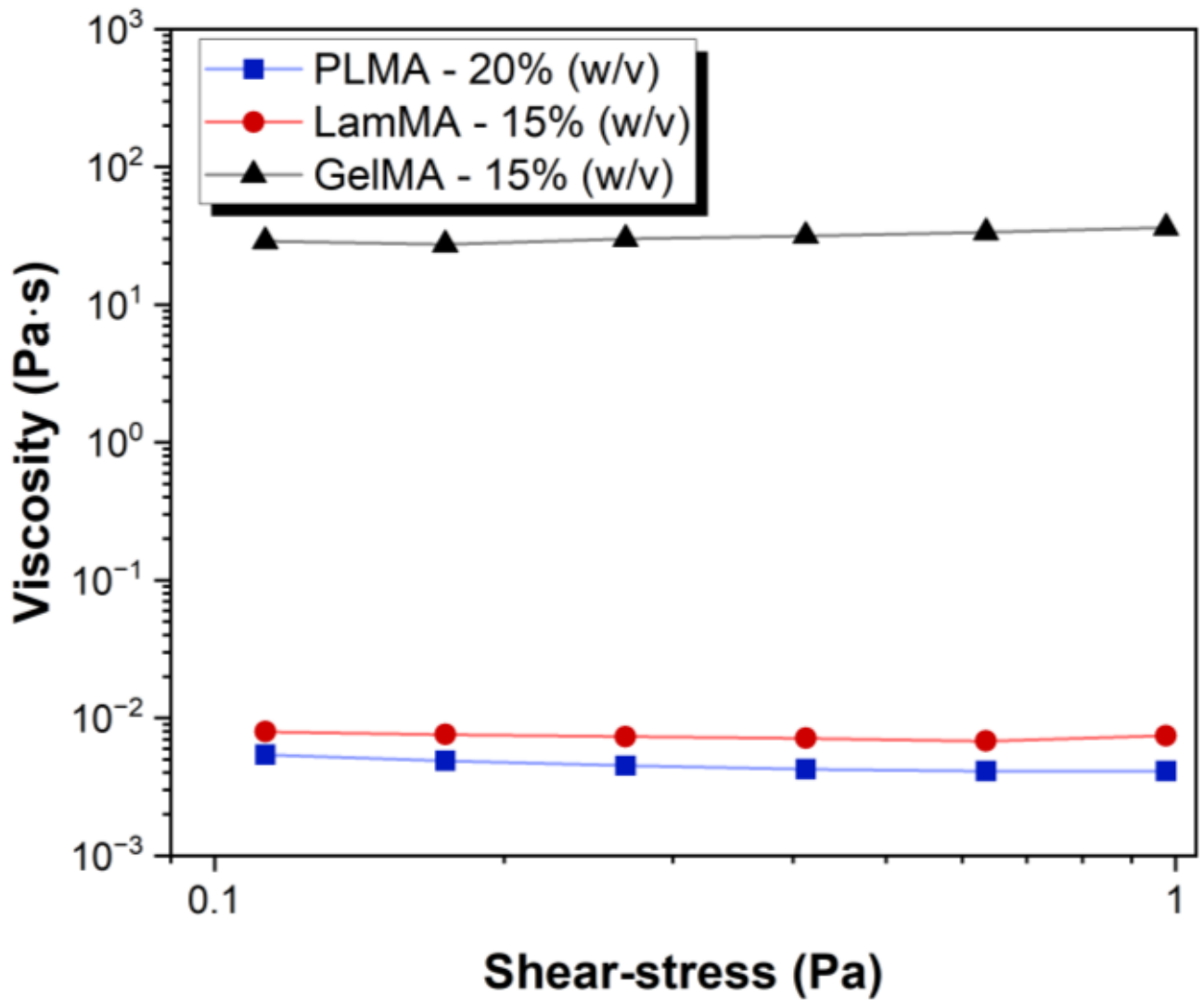

Figure S5: Viscosity of the precursor solutions as a function of applied shear stress. Measurements were performed at 25 °C for all formulations, except GelMA, which was characterized at 30 °C. All precursor solutions exhibited a Newtonian viscosity plateau within the low-shear regime (0.1 – 1 Pa).

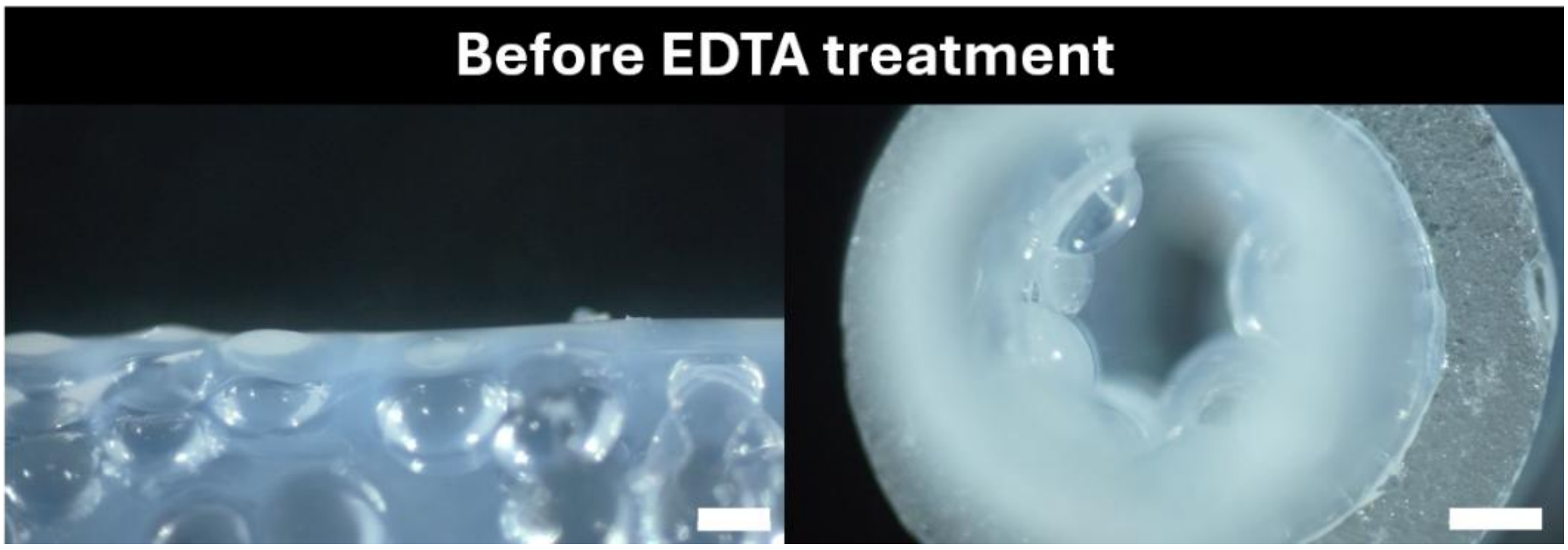

Figure S6. Representative photographs of tubular hydrogels containing embedded alginate-Ca^{2+} beads prior to EDTA treatment. Left: side view (scale bar = 500 µm). Right: top view (scale bar = 1000 µm).

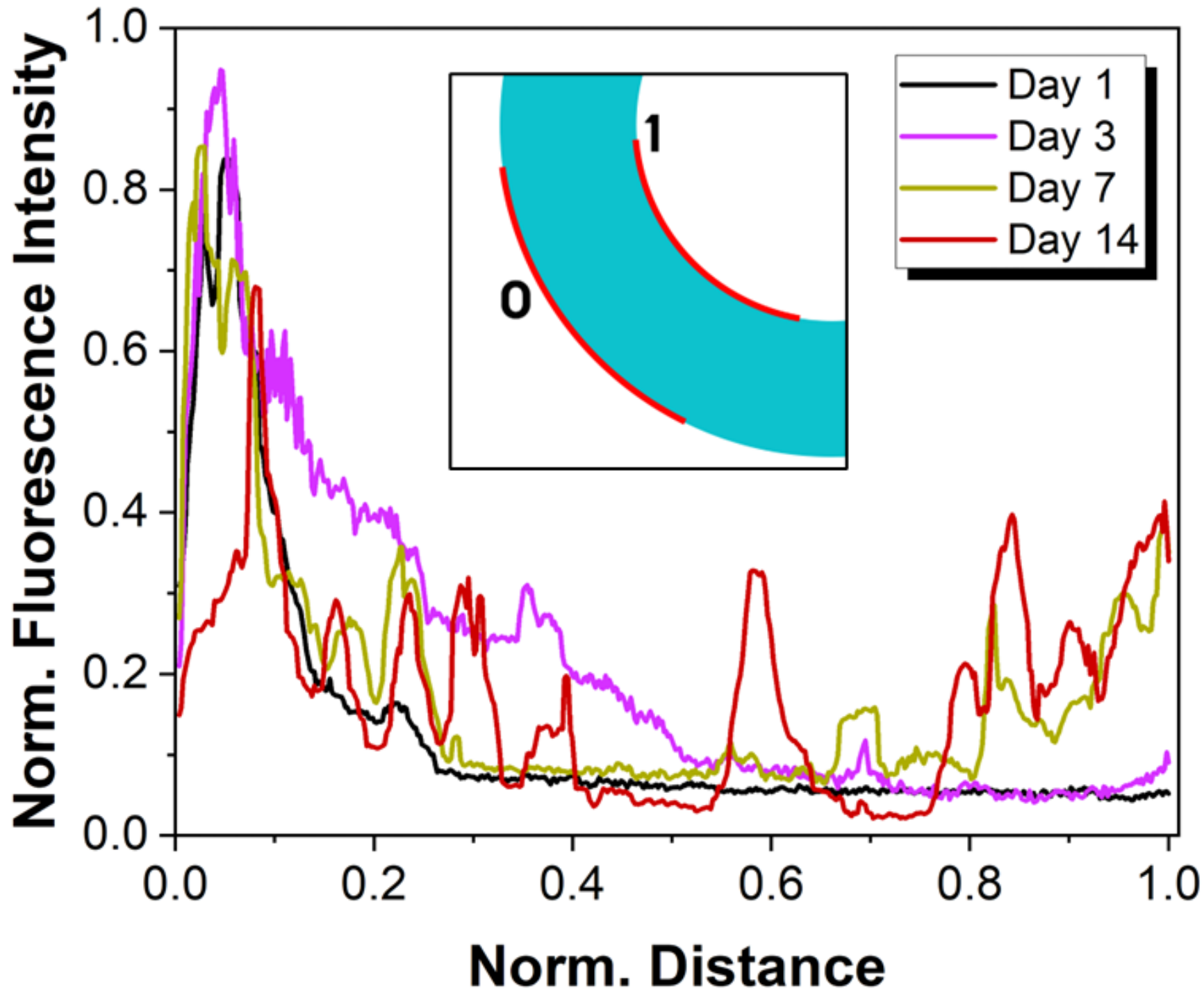

Figure S7: Fluorescence intensity (measured based on Calcein AM-stained cells) was quantified along the wall of PLMA-based tubular hydrogels at multiple time points using ImageJ. Results are shown as normalized distance, with the outer surface of the tubular hydrogel set to 0 and the inner wall set to 1, as illustrated in the inset. Measurements were taken at a minimum of four axial positions per tubular hydrogel.

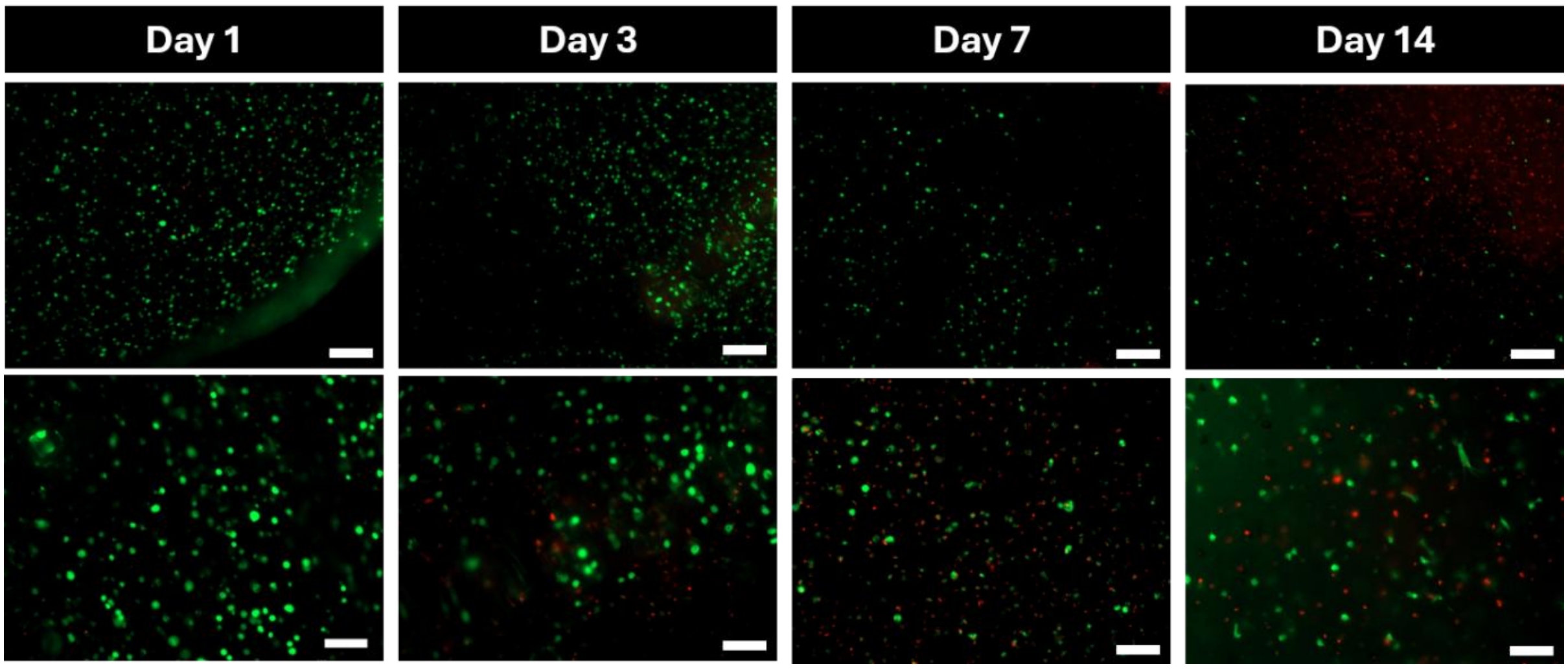

Figure S8: Live/dead fluorescence microscopy images of hASCs encapsulated within PLMA-based bulk hydrogels (control) over 14 days, with Calcein AM (green) indicating live cells and propidium iodide (PI, red) indicating dead cells. Scale bars: 200 μm (top row) and 100 μm (bottom row).

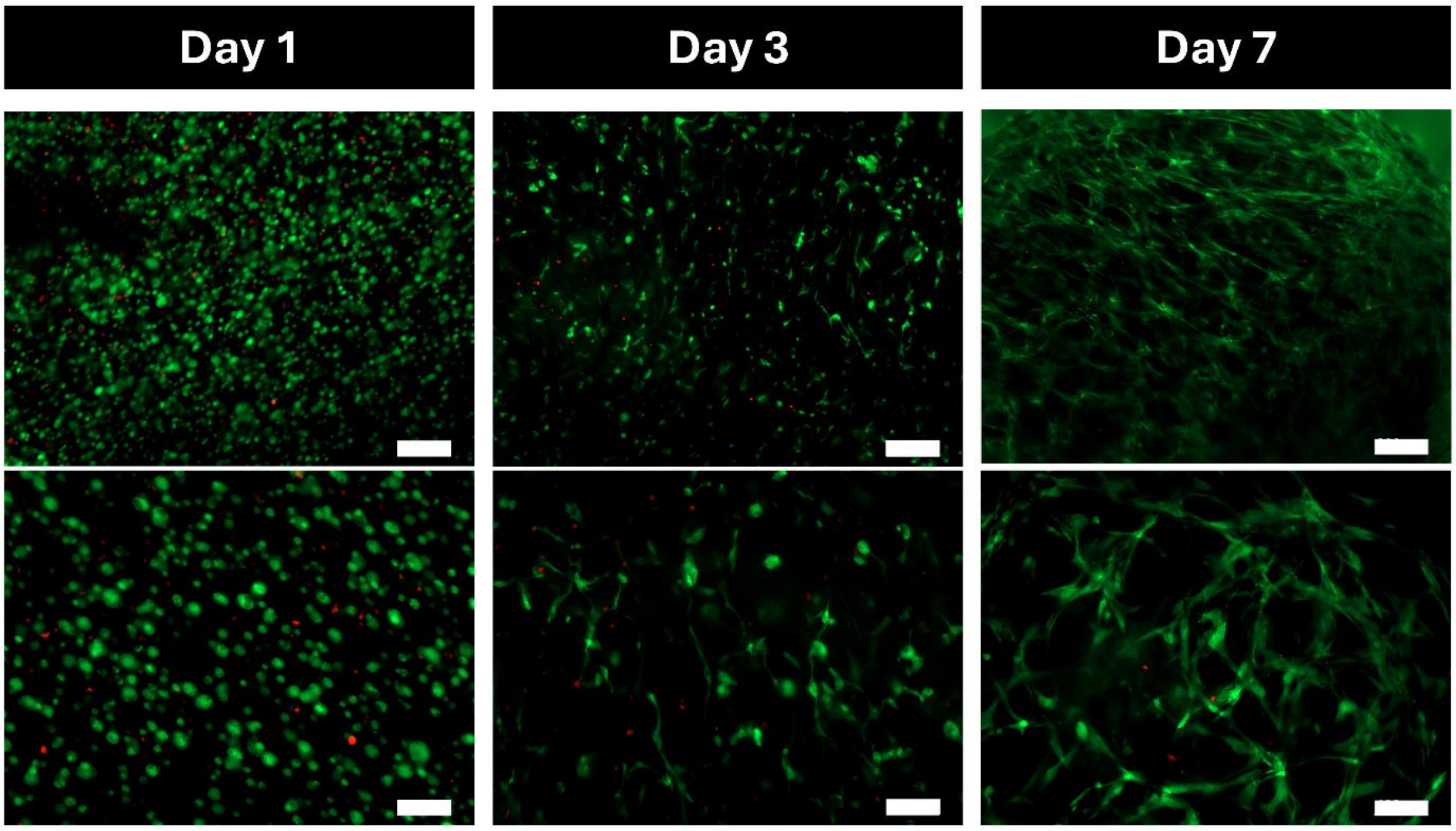

Figure S9: Live/dead fluorescence microscopy images of hASCs/hUVECs encapsulated within PLMA-based hollow tubular constructs over 7 days, with Calcein AM (green) indicating live cells and propidium iodide (PI, red) indicating dead cells. Scale bars: 200 μm (top row) and 100 μm (bottom row).

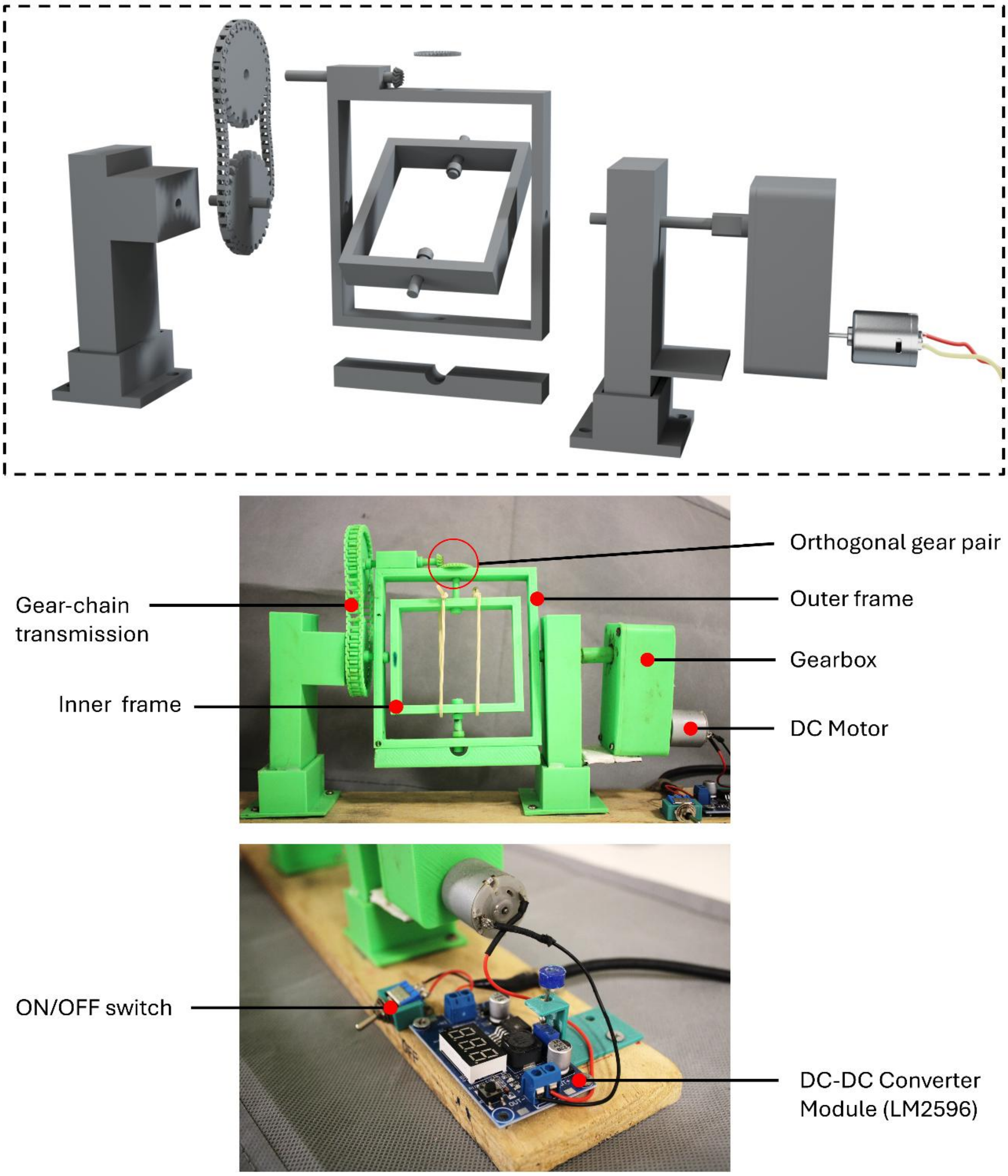

Figure S10: Top: 3D schematic of the biaxial RM apparatus, illustrating the location and assembly of each component. Bottom: Digital photographs of the apparatus from multiple angles, with all components labeled to show the setup and configuration.

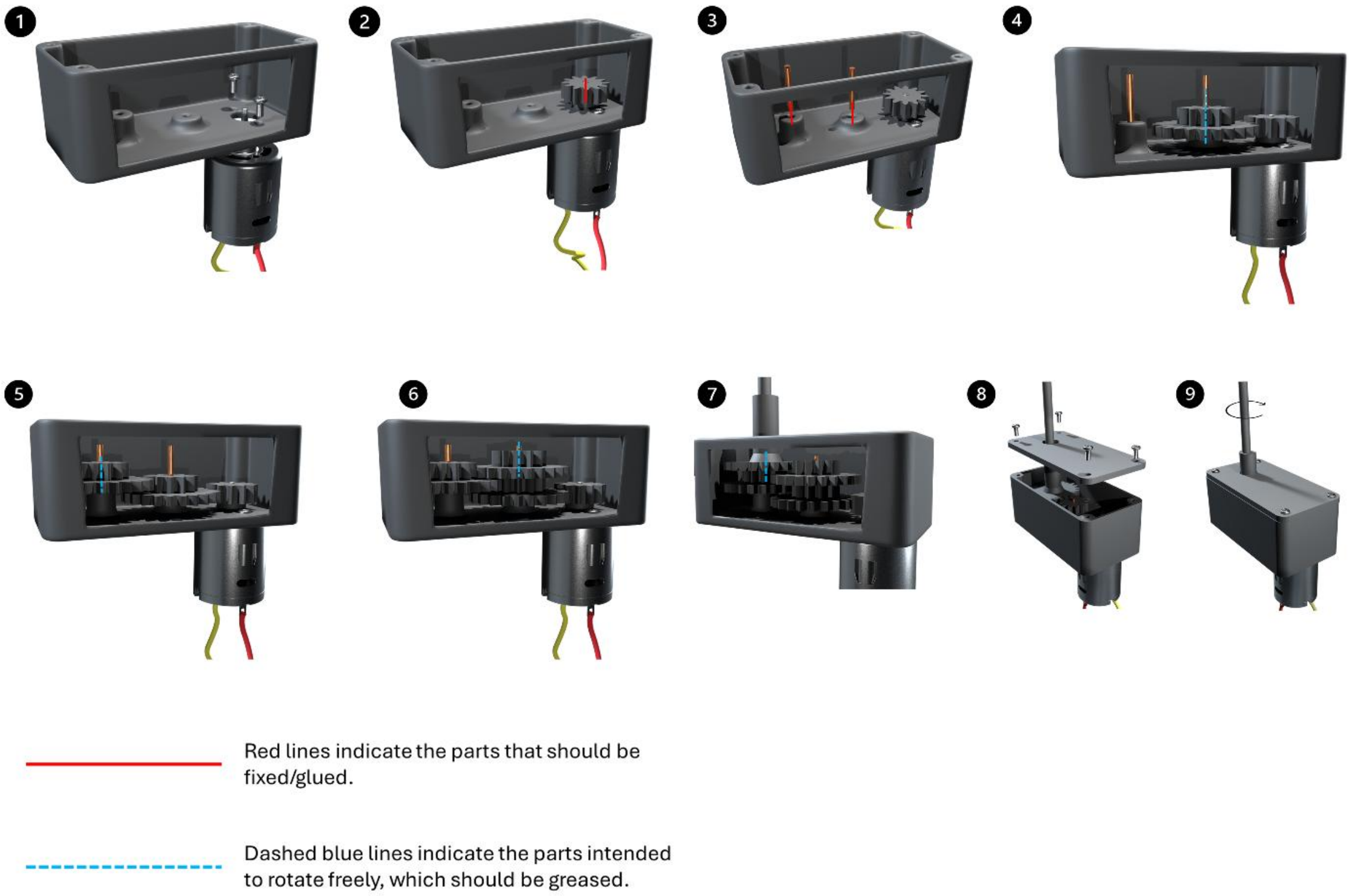

Figure S11: Schematic representation of the gearbox assembly, including multiple views to illustrate how the individual components fit together. Gearbox.stl designed by Tomi Malkki published on https://www.instructables.com/3d-Printed-Gearbox-for-Small-Dc-Motors/ licensed under CC BY-NC-SA 4.0 (https://creativecommons.org/licenses/by-nc-sa/4.0/)

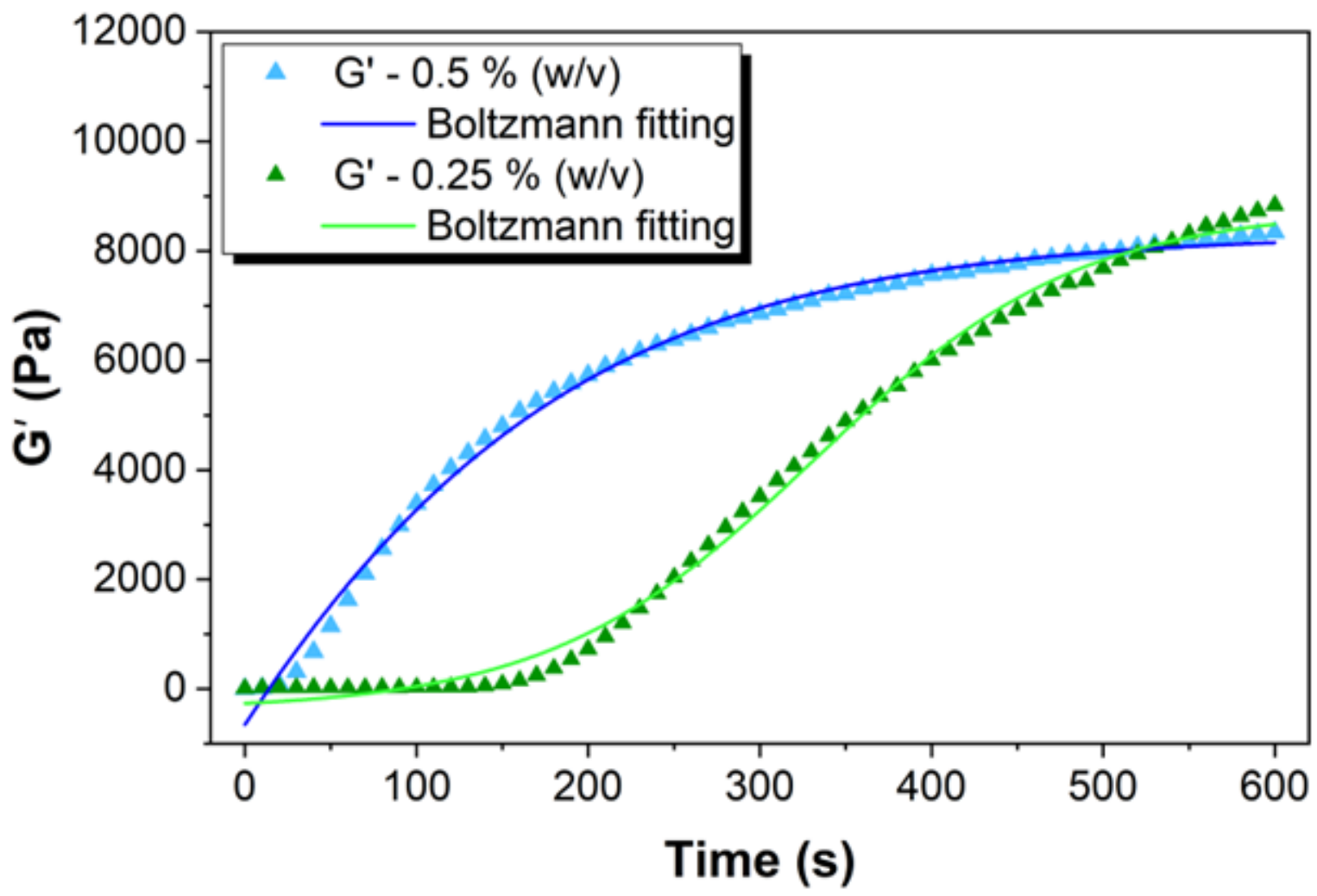

Figure S12: Elastic modulus (G') values obtained from the time-sweep rheological measurements of LamMA at 20% (w/v) with 0.5 % and 0.25 % (w/v) APS/TEMED. The evolution of the G' over time is shown for both formulations, with the corresponding Boltzmann fits overlaid as solid lines.

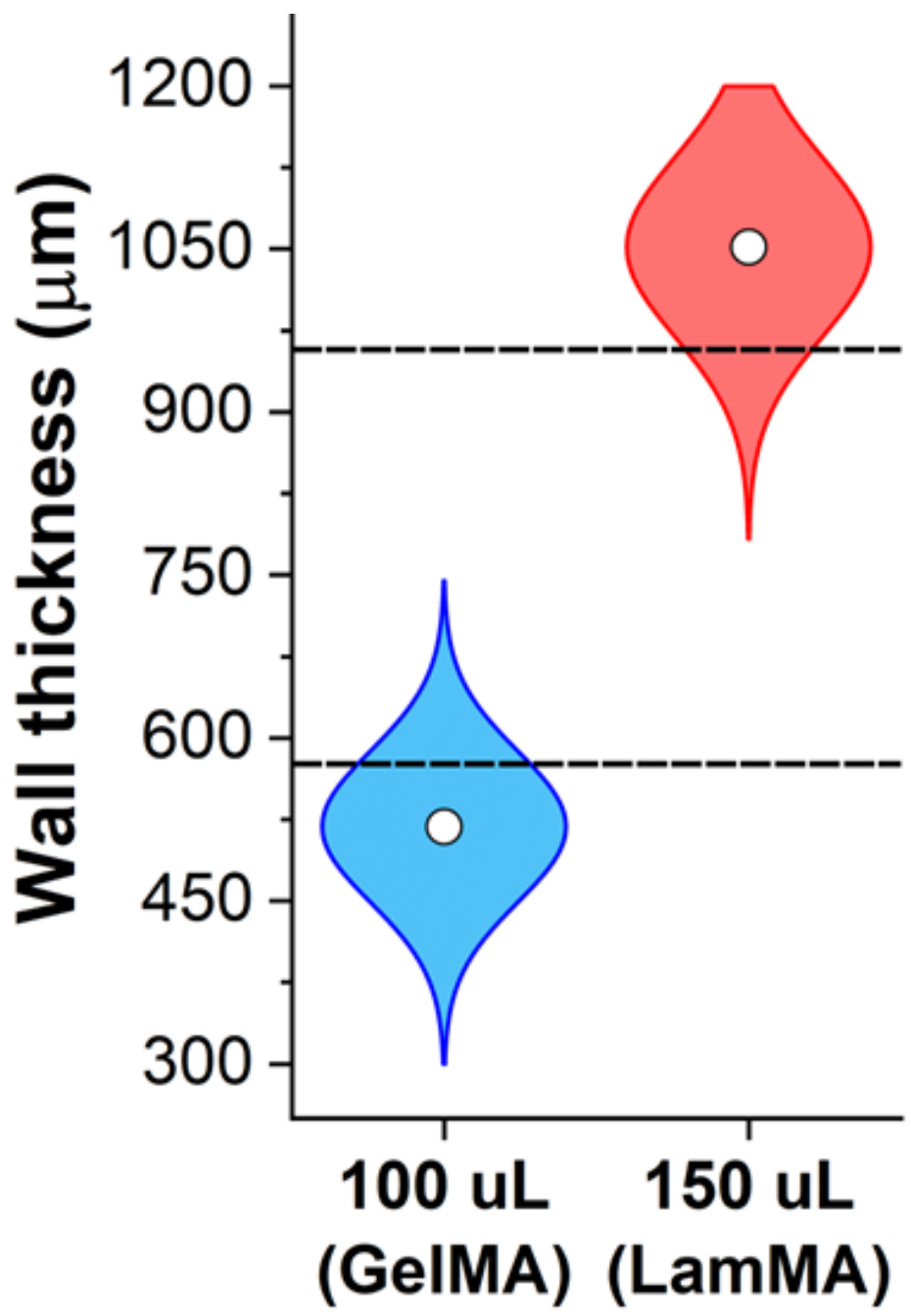

Figure S13: Wall thickness distribution of the LamMA and GelMA hollow spheres. The white circles indicates the average, while the black dashed line represents the predicted wall thickness for the same volume.

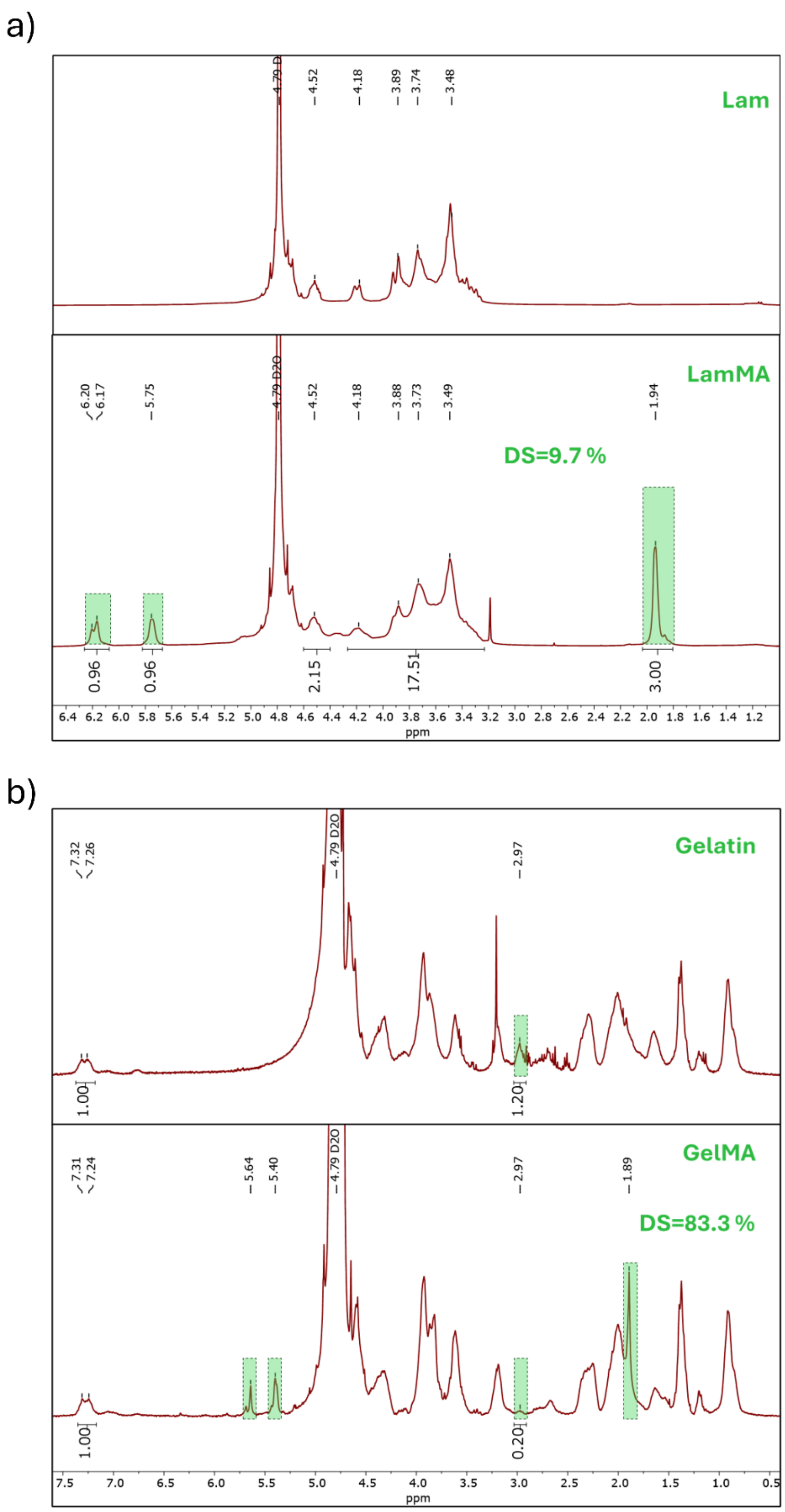

Figure S14: ^{1}H NMR of a) LamMA and b) GelMA. For comparison, the NMR of the native counterparts was also recorded. Spectra were acquired in D_2O at 300.13 MHz using a Bruker Avance-300 spectrometer (Bruker, Germany). Chemical shifts are given in δ (ppm). The degree of substitution (DS) of LamMA was obtained from the integral of the

vinylic protons (δ =5.75 and 6.17 ppm) relative to the polymer backbone region (δ =3.20-4.60 ppm) [1]. The DS of GelMA was obtained from the decrease of lysine methylene proton integrals (δ ≈ 2.97 ppm) relative to gelatin, calculated as $1-(I_{\text{GelMA}}/I_{\text{gelatin}})$ after normalization to the phenylalanine aromatic region (δ ≈ 7.25–7.35 ppm)[2]. The methacrylate-related signals are highlighted in green in both spectra.

Table S1: Parameters obtained from Boltzmann fitting of time-sweep rheological measurements of 20% (w/v) LamMA with 0.5 and 0.25% (w/v) APS/TEMED. The maximum slope reflects the rate of gelation, with higher values corresponding to faster network formation.

	**0.5 % (w/v)**	**0.25 % (w/v)**
A1	-26822.2 ±13911.1	-387.4 ± 82.4
A2	8315.9 ± 85.5	8761.0 ± 112.6
x_0	-149.9 ± 83.7	331.7 ± 3.0
dx	140.0 ± 11.4	77.2 ± 3.1
r^2	0.995	0.996
Slope*	62.75	29.63

* maximum slope obtained from the derivative of the Boltzmann function at its inflection point (x_0) using the calculated dx.

References:

1. Castanheira, E.J., et al., *In-Bath 3D Printing of Anisotropic Shape-Memory Cryogels Functionalized with Bone-Bioactive Nanoparticles*. ACS Applied Materials & Interfaces, (2024). 16(15): p. 18386-18399, DOI: 10.1021/acsami.3c18290.
2. Hoch, E., et al., *Chemical tailoring of gelatin to adjust its chemical and physical properties for functional bioprinting.* Journal of Materials Chemistry B, (2013). 1(41): p. 5675-5685, DOI: 10.1039/C3TB20745E.
